# Matrix Stiffness Induces Endothelial Network Senescence

**DOI:** 10.1002/advs.76304

**Published:** 2026-07-02

**Authors:** Jiyeon Song, Alexandra N. Rindone, Ya Guan, Connor D. Amelung, Prarthana Sanjay Daswani, Jennifer H. Elisseeff, Sharon Gerecht

**Affiliations:** ^1^ Department of Biomedical Engineering Duke University Durham North Carolina USA; ^2^ Translational Tissue Engineering Center Wilmer Eye Institute and Department of Biomedical Engineering Johns Hopkins University Baltimore Maryland USA; ^3^ Bloomberg–Kimmel Institute for Cancer Immunotherapy Sidney Kimmel Comprehensive Cancer Center School of Medicine Johns Hopkins University Baltimore Maryland USA; ^4^ Department of Chemical and Biomolecular Engineering Johns Hopkins University Baltimore Maryland USA

**Keywords:** biomaterial, cell biology, endothelial stem cell, engineered tissue, extracellular matrix, fibrosis, hydrogel scaffold, notch signaling pathway, phenotype, senescence

## Abstract

Identifying the drivers of cellular senescence that contribute to the decline in vascular function with age and disease is critical for developing restorative interventions. Here, we investigated how increased mechanical stress from extracellular matrix (ECM) stiffening shapes endothelial cell (EC) senescence. We developed a 3D human in vitro model that decouples mechanical stress from inflammatory or biochemical signals, enabling the study of senescence responses to tissue stiffening alone. We found that matrix stiffening induces an EC senescence phenotype with elevated p16/p21 and an immunomodulatory senescence‐associated secretory phenotype (SASP), in the absence of inflammatory signals. This mechano‐induced senescence activates Notch signaling, and treatment with an FDA‐approved γ‐secretase inhibitor attenuates stiffness‐induced senescence. Analysis of fibrotic capsule tissue from patients with synthetic breast implants, a model of localized, mechanically driven fibrosis, validated an increase in p16^+^Notch1^+^ endothelial populations. Complementary single‐cell RNA sequencing data further confirmed enrichment of Notch‐ and SASP‐related gene programs. Our work provides a human‐relevant platform for studying targetable stages of endothelial mechanoaging and identifies potential therapeutic targets associated with stiffness‐induced endothelial senescence for mechanically remodeled tissues.

## Graphical abstract

Using a 3D human in vitro model that decouples mechanical stress from inflammatory or biochemical signals, matrix stiffening induces a senescence phenotype in endothelial networks. This mechano‐induced senescence activates Notch signaling, and pharmacologic Notch inhibition attenuates this stiffness‐induced senescence.

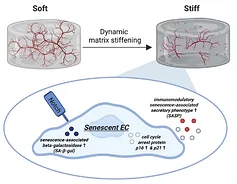

## 1 Introduction

Endothelial cell (EC) senescence has been recognized as a contributor to vascular aging and dysfunction, including impaired barrier integrity, chronic inflammation, and tissue remodeling [1]. Senescent ECs undergo persistent cell‐cycle arrest and develop a senescence‐associated secretory phenotype (SASP) that disrupts vascular homeostasis and influences surrounding stromal and immune cell behavior [2, 3]. While biochemical stressors, including oxidative stress, DNA damage, and inflammatory mediators, are established inducers of EC senescence, mechanical changes in the extracellular microenvironment have also emerged as important regulators of endothelial behavior through mechanosensing and mechanotransduction pathways.

Progressive matrix stiffening is a characteristic feature of aging and fibrotic tissue, arising from increased matrix deposition and cross‐linking that alter the mechanical cues perceived by surrounding cells [4]. In the microvasculature, ECs directly interface with the vascular basement membrane and perivascular matrix, placing them in immediate contact with these biomechanical cues [5]. Matrix stiffness is therefore an important component of the endothelial niche [6, 7, 8]. However, its role in regulating EC senescence in a 3D microvascular network remains incompletely understood.

A key enabler of this work is a hydrogel system designed to exert mechanical control over extracellular matrix (ECM) stiffening around 3D lumenized vascular networks. We developed a tunable hydrogel platform that simulates the dynamic increase in ECM stiffness to investigate how mechanical forces regulate EC network senescence. This hydrogel system decouples mechanical stimuli from inflammatory or biochemical signals, which are known to regulate cellular senescence. Unlike the adherent culture system, this approach offers a physiologically relevant framework for examining how dynamic matrix stiffening regulates endothelial senescence in a 3D microvascular context.

The engineered system captured a mechanically induced senescence state in endothelial networks, marked by elevated senescence marker expression and a non‐canonical, immunomodulatory SASP distinct from the classical IL‐6/IL‐8‐enriched profile. In these stiffened conditions, Notch signaling was associated with the senescent endothelial response. Pharmacological inhibition of Notch signaling reduced endothelial network senescence. Validation of the model with fibrotic capsule tissue from human breast implants and accompanying single‐cell RNA sequencing data revealed an enrichment of Notch1^+^ senescent EC populations in stiffened environments, supporting the translational relevance of our in vitro findings. This work establishes a mechanosensitive platform to dissect biomechanical inputs and identify targetable drivers of endothelial senescence.

## 2 Results

### 2.1 Hydrogel Platform to Study Stiffness‐Induced Endothelial Network Senescence In Vitro

To examine the role of matrix stiffness in EC network senescence, we established a physiologically relevant 3D model that supports the formation of lumenized endothelial networks in a soft collagen‐rich matrix, followed by on‐demand stiffening during cell culture under controlled conditions. This two‐step cross‐linking strategy recapitulates the increase in ECM stiffness, while holding biochemical cues constant, thereby isolating mechanics as the independent variable [8]. Unlike in vivo, where stiffness changes coincide with shifts in pathological soluble factors, our system enables selective interrogation of mechanical effects on EC network senescence.

**FIGURE 1:**
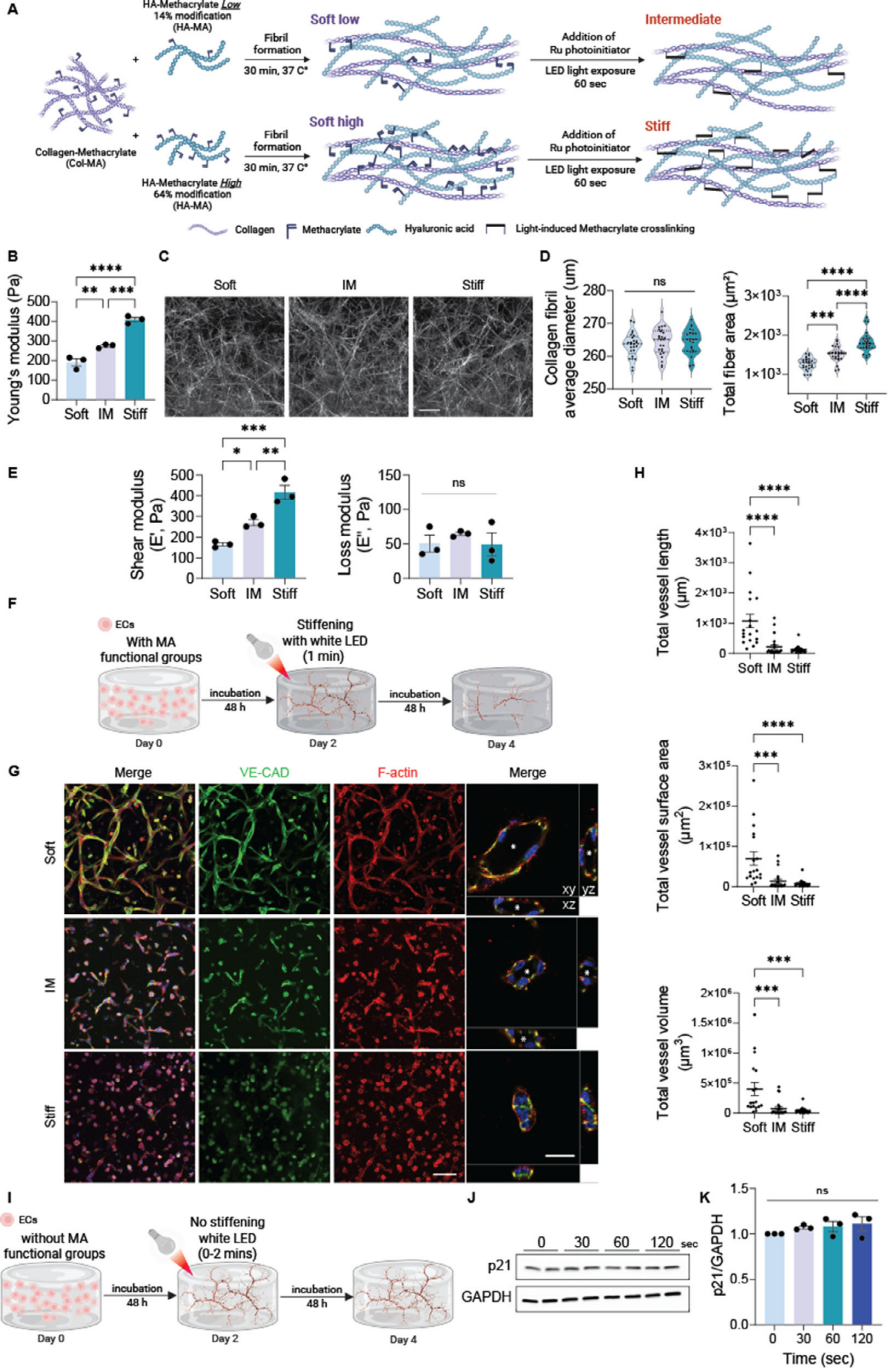
In vitro hydrogel system to model endothelial network senescence. (A) Schematic of the Col‐MA/HA‐MA hydrogel system using two different degrees of methacrylate incorporation on HA. (B) Young's modulus of Soft (high), Intermediate (IM), and Stiff hydrogels. N = 3. (C) Representative reflective confocal images of collagen fiber structures at varying stiffness levels. Scale bar = 20 µm. (D) Quantification of collagen fibril average diameter and total fiber area. N = 5 with 5 fields of images in each. (E) Microscale viscoelastic characterization measured by AFM‐based microrheology, including shear modulus and loss modulus, in Soft, IM, and stiff hydrogels. (F) Schematic of the 3D stiffening hydrogel system. ECs were encapsulated in Col‐HA hydrogels and allowed to form networks for 48 h, followed by photo‐crosslinking using a ruthenium photoinitiator for 1 min. Both soft control and stiffened hydrogels were subsequently cultured for an additional 48 h prior to analysis. (G) Representative maximum intensity projections of confocal z‐stack showing microvascular networks and orthogonal cross‐sections of 3D lumina (asterisks) in Soft, IM, and stiff hydrogels. Increased stiffness reduced vessel formation, as quantified in (H) by total vessel length, surface area, and volume. Each biological replicate (N = 3) included two independent hydrogel samples, with ≥ 2 fields of view per sample analyzed. DAPI in blue, VE‐Cad in green, F‐actin in red. The scale bars: 100 µm (networks) and 25 µm (lumen cross‐sections). (I) Schematic of control experiment assessing the effect of radical exposure in the absence of matrix stiffening. ECs were encapsulated in unmodified collagen hydrogels lacking MA functional groups and incubated for 48 h. On Day 2, 3D cell‐laden hydrogel constructs were then exposed to white LED light (0–120 s) in the presence of photoinitiator, followed by continued culture to Day 4. (J) Western blot analysis and (K) quantifications of p21 levels in ECs encapsulated in unmodified collagen hydrogels (no stiffening) to evaluate the effect of radical exposure on cellular senescence. p21 levels were normalized to GAPDH. N = 3 with two technical replicates per biological replicate. Statistical analysis was performed using one‐way ANOVA followed by Tukey's multiple comparisons test. Data are presented as mean ± SEM. Significance levels were set at *p ≤ 0.05, **p ≤ 0.01, ***p ≤ 0.001, and ****p ≤ 0.0001.

We previously established a methacrylated collagen (Col‐MA) and hyaluronic acid (HA‐MA) platform in which collagen first forms a physically cross‐linked gel at 37°C, followed by ruthenium‐mediated, light‐induced polymerization of methacrylate groups [8]. In this first‐generation system [8], increasing visible light exposure to tune gel stiffness also elevated the flux of photogenerated radicals and reactive oxygen species (ROS) byproduct, confounding mechanical effects with ROS‐driven senescence [9]. To overcome this limitation, we developed a second‐generation Col‐MA/HA‐MA system that exhibits different stiffness levels under the same photochemical conditions. Collagen I was retained as the bioactive scaffold for structural and mechanotransducive cues [10, 11]. Hydrogel stiffness was modulated by varying the degree of HA methacrylation (14 and 64 mol%; Figure S1) while holding light intensity, duration, and photoinitiator constant. Importantly, all hydrogel formulations were prepared with identical concentrations of collagen I and hyaluronic acid, thereby maintaining consistent ligand density while modulating stiffness through differences in HA methacrylation. Under these matched conditions, 14 mol% HA‐MA yielded an intermediate (IM) stiffness, whereas 64 mol% produced a stiffer matrix, while the Soft low and high formulations prepared with different HA‐MA variants had comparable moduli (Figure 1A; Figure S2A).

Oscillatory rheometry measurements confirmed effective stiffness separation, with Young's modulus ∼2.28 fold higher in Stiff and ∼1.43 fold higher in IM relative to Soft (Figure 1B), closely mimicking the change in ECM stiffening in aging mouse tissue compared to young [8]. Equilibrium swelling ratios were 397.01 ± 47.68, 254.62 ± 46.92, and 184.19 ± 7.62 for Soft, IM, and Stiff, respectively (Figure S2B). Reflective confocal imaging revealed unchanged collagen fibril width across groups (263–264 nm) but increased total collagen fiber area with stiffness (Figure 1C,D), consistent with higher cross‐link density rather than changes to the intrinsic collagen/HA network structure. The two Soft formulations also exhibited comparable swelling ratios, fibril widths, and fiber areas, indicating differences in HA methacrylation did not alter hydrogel properties prior to matrix stiffening (Figure S2B–E).

To further evaluate whether stiffness modulation introduced microscale alterations in the collagen‐based fibrillar network, we performed viscoelastic measurements using oscillatory atomic force microscopy (AFM)‐based microrheology [12]. The storage modulus (E′) showed trends consistent with bulk stiffness measurement, while the loss modulus (E″) remained comparable across Soft, IM, and Stiff conditions (Figure 1E). These results indicate that stiffness was selectively tuned, while viscous dissipation and microscale fiber rearrangement were preserved. The two Soft hydrogel formulations also showed comparable storage and loss moduli (Figure S2F,G).

We next encapsulated endothelial colony‐forming cells (ECFCs) in the hydrogels and allowed them to form microvascular networks for 48 h. Matrix stiffening was then induced in situ with a photoinitiator and LED illumination, followed by an additional 48 h of culture (Figure 1F). Quantitative Live/Dead analysis found over 88% cell viability across all hydrogel conditions, confirming the cytocompatibility of the dynamic stiffening platform (Figure S2H,I). During the post‐stiffening period, hydrogels exhibited around 10% mass loss over 48 h with no significant differences across conditions (Figure S2K).

To determine whether matrix stiffening induces microvascular regression with aging [8], we evaluated microvascular network morphology and luminal structure. In both the IM and Stiff conditions, lumenized endothelial networks exhibited disrupted morphology, characterized by constricted and disorganized luminal structures, along with significantly reduced total vessel length, surface area, and volume (Figure 1G,H). This indicates that matrix stiffening impairs the structural integrity of microvascular networks. By contrast, networks in Soft gels remained intact and continued to mature, highlighting stiffness‐dependent effects on vascular phenotype and aligning with our previous observation [8]. Consistently, both Soft formulations without light exposure exhibited comparable microvessel formation, confirming that different levels of methacrylate functionalization did not affect microvasculature formation capacity (Figure S2L,M). Overall, no differences were detected in the material properties and EC network assembly between the two soft hydrogel formulations (Table S1). Consequently, we used the soft gel with higher MA modification in the HA as the control in all our experiments. Additionally, since both IM and Stiff hydrogels are polymerized under the same photoinitiation conditions and precursor concentrations (Table S1), any variations in cellular responses can be attributed to matrix stiffness rather than radical exposure or ligand density. This dynamic stiffening system effectively isolates matrix stiffness as the only variable, allowing us to study EC senescence due to mechanics rather than ROS generated during photopolymerization.

To further ensure that senescence outcomes reflected mechanics rather than radical exposure, we tested whether LED illumination and photoinitiator‐derived radicals alone induce EC senescence in the absence of matrix stiffening. We encapsulated ECs in unmodified collagen hydrogels lacking methacrylate functional groups, which therefore do not undergo stiffening upon light exposure even in the presence of photoinitiator, and the 3D EC‐laden constructs were exposed to light for 0–120 s, spanning durations both shorter and longer than our standard stiffening condition (Figure 1I). p21 senescence marker expression was unchanged across all exposure times (Figure 1J,K), indicating that neither light exposure nor photoinitiator‐derived radical generation alone induces p21 expression in the absence of matrix stiffening. These findings support the use of the non‐illuminated Soft hydrogel condition as the control group and indicate that any subsequent senescence‐associated responses observed in the stiffened hydrogel system can be attributed to matrix stiffening rather than photochemical exposure.

### 2.2 Stiffening Matrix Induces Endothelial Network Senescence

**FIGURE 2:**
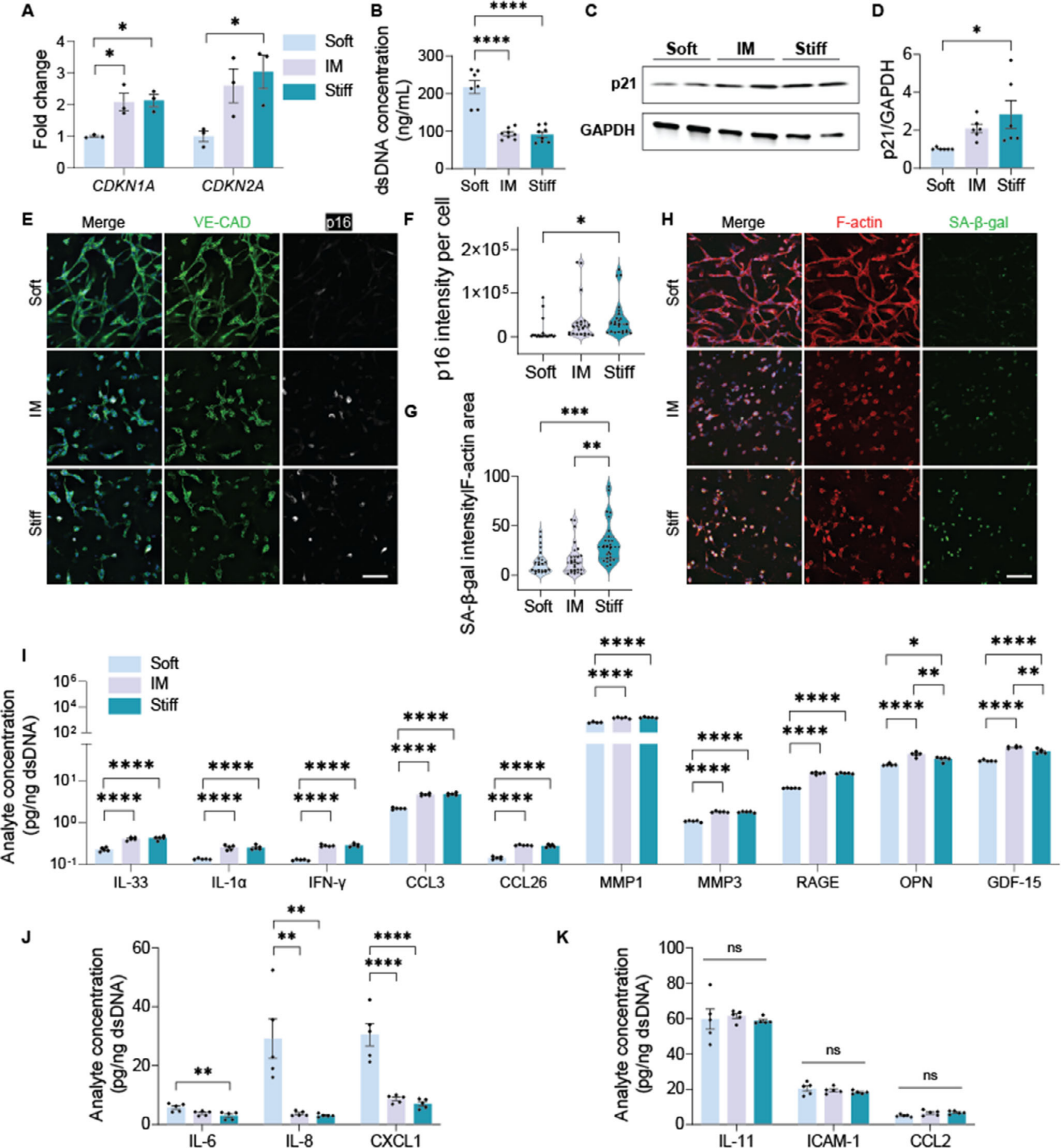
Matrix stiffness‐induced endothelial network senescence. Endothelial networks cultured in hydrogels of increasing stiffness show elevated expression of senescence markers: (A) *CDKN1A* and *CDKN2A* mRNA levels measured by qRT‐PCR. (B) Day 4 dsDNA content quantified by PicoGreen assay, showing reduced cell number under stiffened conditions. Each biological replicate (N = 2) included three to four independent hydrogel samples, with one data point representing a single hydrogel sample. (C, D) p21 protein levels assessed by Western blot, and (E, F) p16 protein expression visualized by immunofluorescence staining. Representative confocal images show microvascular networks stained for VE‐Cadherin in green, p16 in white, and nuclei in blue. Scale bar is 100 µm. (G, H) SA‐β‐gal activity increased with matrix stiffening. Representative confocal images show networks stained for F‐actin (red), SA‐β‐gal (green), and nuclei (blue). Scale bar: 100 µm. For all immunofluorescence‐based analyses (E–H), each biological replicate (N = 3) included two independent hydrogel samples, with ≥ 3 fields of view per sample. (I–K) Senescence‐associated secretory phenotype (SASP) factors, including cytokines, chemokines, and MMPs, analyzed using a Luminex assay. Values were normalized to the dsDNA content reported in (B). Each biological replicate (N = 2) included two to three independent hydrogel samples. The complete dataset of all analytes measured in the panel is provided in Tables S2 and S3. Statistical analysis was performed using one‐way ANOVA followed by Tukey's multiple comparisons test. Data are presented as mean ± SEM. Significance levels were set at ns = not significant (*p* > 0.05), *p ≤ 0.05, **p ≤ 0.01, ***p ≤ 0.001, and ****p ≤ 0.0001.

Next, we tested whether dynamic matrix stiffening drives endothelial network senescence. Following 48 h of microvascular network formation, lumenized endothelial networks were exposed to dynamically stiffened matrix environments corresponding to IM and Stiff conditions for an additional 48 h (Figure S3). At the transcriptional level, the senescence markers *CDKN1A* (p21) and *CDKN2A* (p16) were significantly upregulated with increasing matrix stiffness, with the highest expression level observed in Stiff gels (Figure 2A). In line with upregulation of *CDKN1A* and *CDKN2A*, Day 4 dsDNA content and Hoechst^+^ nuclei counts were significantly reduced in IM and Stiff conditions compared to Soft gels (Figure 2B, Figure S1J), indicating fewer cells at the experimental endpoint despite identical initial seeding densities. This reduction in cell number, combined with maintained overall viability as assessed by Live/Dead analysis (Figure S2H,I), suggests reduced proliferative expansion rather than increased cell death under stiffened conditions. Western blot analysis further showed a stiffness‐dependent increase in p21 protein expression (Figure 2C,D). Consistent with these findings, immunofluorescence analysis revealed elevated p16 expression and increased SA‐β‐gal activity in Stiff matrices (Figure 2E–H).

In addition to upregulating senescence markers, dynamic matrix stiffening induced a distinct senescence‐associated secretory phenotype (SASP). To characterize this mechanoaging‐associated SASP, we analyzed culture supernatants for cytokines, chemokines, and proteases secreted by senescent ECs in the absence of exogenous inflammatory stimulation using a multiplex Luminex panel (full dataset provided in Tables S2 and S3). Matrix stiffening significantly increased secretion of several immunomodulatory alarmins and stress‐associated factors, including IL‐33, IL‐1α, IFN‐γ, CCL3, CCL26, MMP1, and MMP3 (Figure 2I). Notably, IL‐1α is an EC‐senescence cue that coordinates cytokine signaling networks [13]. Stiffened conditions also elevated RAGE, OPN, and GDF‐15, senescence‐associated biomarkers linked to vascular aging and cellular stress responses (Figure 2I) [14, 15, 16]. In contrast, canonical pro‐inflammatory SASP cytokines such as IL‐6, IL‐8, and CXCL1 were significantly suppressed under stiffened conditions (Figure 2J), while IL‐11, CCL2, and ICAM‐1 levels remained unchanged across varying matrix stiffness (Figure 2K). Furthermore, several classical inflammatory mediators, including TNF‐α, IL‐17, IL‐13, VCAM‐1, and TGF‐α, were consistently below the assay detection limit across all conditions, while IL‐1β, CXCL2, and CCL8 showed inconsistent detection across replicates (Tables S2 and S3). These findings suggest that matrix stiffening induces a selective endothelial SASP enriched in alarmin‐ and stress‐associated factors, accompanied by suppression of canonical pro‐inflammatory cytokines.

To further characterize remodeling‐associated responses in stiffness‐induced senescent ECs, we examined the expression of matrix remodeling enzymes and ECM‐related genes by qPCR (Figure S4). *MMP9* was downregulated under stiffened conditions, suggesting reduced MMP9‐dependent angiogenic remodeling capacity [17]. In contrast, *CYR61* expression, which contributes to endothelial survival signaling [18], remained relatively stable despite vascular network regression observed under stiffened conditions. Matrix stiffening increased expression of the basement membrane‐associated genes *MMP2* and *COL4A1* while reducing expression of fibrotic ECM‐related genes, including *CTGF*, *COL3A1*, *FN1*, and *LOX*, during the early response to mechanical stimulation.

Collectively, these findings demonstrate that matrix stiffening drives a senescence program in microvascular networks, accompanied by an alarmin‐ and stress‐associated SASP and basement membrane remodeling alterations [19, 20]. Given these coordinated responses following matrix stiffening, we next investigated whether mechanosensitive signaling pathways contribute to stiffness‐induced EC senescence.

### 2.3 Matrix Stiffening Activates Notch Signaling, Leading to Endothelial Network Senescence

To investigate signaling pathways underlying our stiffness‐induced senescence program, we next examined Notch signaling, a pathway that regulates endothelial cell fate, adherens junction organization, and vascular homeostasis in response to microenvironmental cues [21]. Matrix stiffness has been shown to directly modulate ligand‐dependent Notch signaling activity in ECs [22], while matrix rigidity and cytoskeletal tension regulate Notch ligand expression through mechanotransduction pathways [23]. Furthermore, Notch activation has been associated with EC senescence, characterized by p21, p16, and SA‐β‐gal induction, in both replicative aging and pathological vascular contexts [24, 25]. Building on our previous finding that dynamic matrix stiffening induces focal adhesion kinase activation and junctional restructuring in 3D endothelial networks [8], we hypothesized that matrix stiffening may engage Notch signaling as part of the mechanotransductive response driving EC senescence.

**FIGURE 3:**
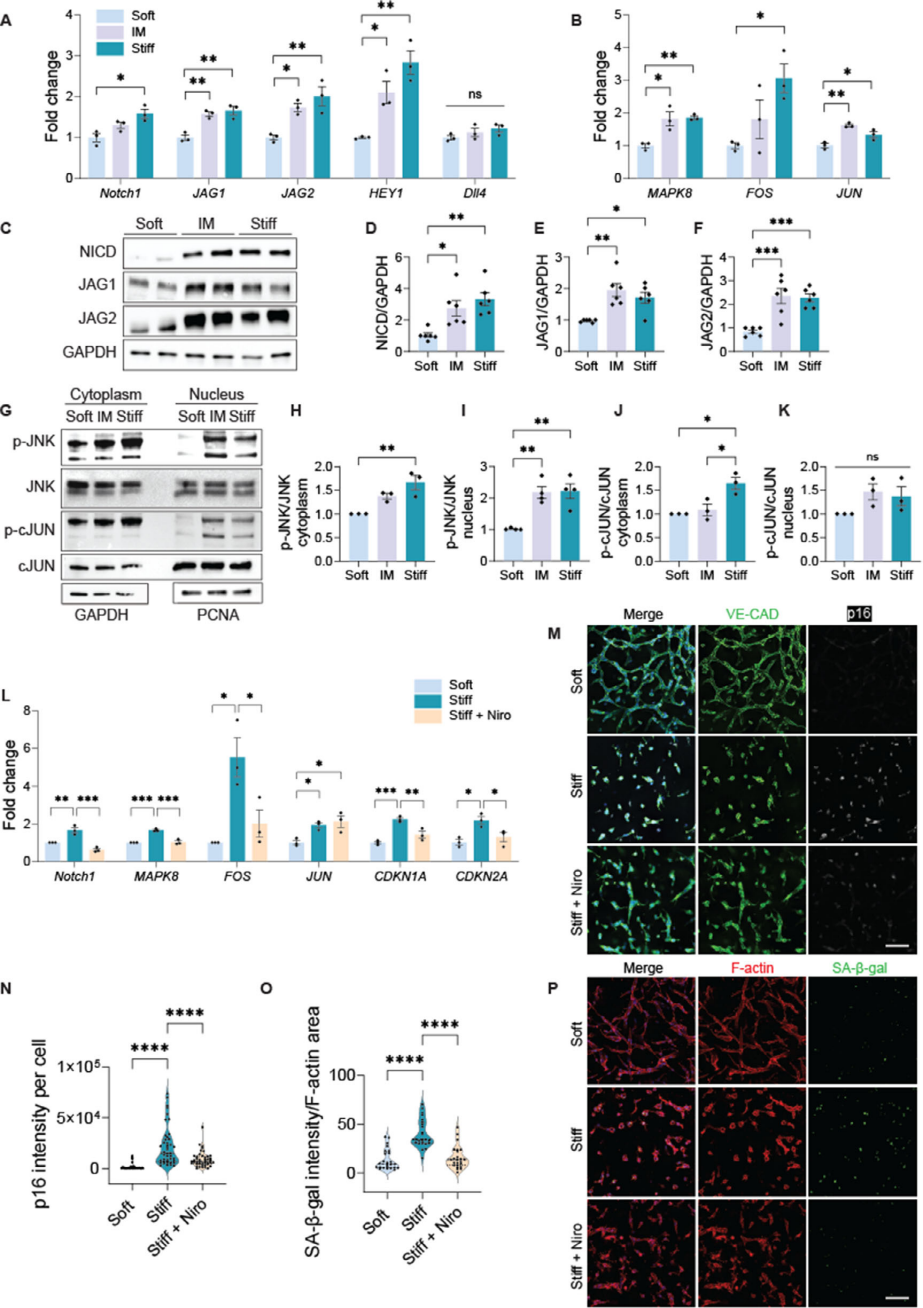
Matrix stiffening activates Notch to drive JNK‐FOS signaling and endothelial senescence. (A) qRT‐PCR analysis revealed significant upregulation of *Notch1* receptor, ligands *JAG1* and *JAG2*, and downstream target *HEY1* under stiff conditions. *Dll4* ligand gene expression remained unchanged upon matrix stiffening. N = 3. (B) Matrix stiffening also induced *MAPK8* (encoding JNK1), *FOS*, and *JUN* gene expression, key components of the JNK‐AP‐1 signaling axis, as measured by qRT‐PCR. N = 3. (C–F) Western blot analysis confirmed increased protein levels of cleaved‐Notch1 (NICD), JAG1, and JAG2 in stiffened matrices. GAPDH was used as a protein loading control. N = 3. (G–K) Matrix stiffening increased phosphorylation of JNK (p‐JNK) in both cytoplasmic and nuclear fractions (N = 4), while phosphorylation of c‐JUN (p‐c‐JUN) was elevated only in the cytoplasm (N = 3). This suggests that nuclear p‐c‐JUN activation is independent of stiffness‐mediated Notch signaling. GAPDH and PCNA were used as protein loading controls for the cytoplasm and nucleus, respectively. (L) Treatment with nirogacestat (Niro), a γ‐secretase inhibitor, reduced stiffness‐induced expression of *Notch1*, *MAPK8*, *FOS*, *CDKN1A*, and *CDKN2A* (N = 3), suggesting that Notch signaling contributes to JNK‐AP‐1 activation and senescence‐associated cell cycle arrest under matrix stiffening. (M, N) Immunofluorescence staining exhibited reduced p16 expression in nirogacestat‐treated microvascular networks compared to untreated stiff controls. Representative confocal images show endothelial networks stained for VE‐Cadherin in green, p16 in white, nuclei in blue. (O, P) Nirogacestat treatment suppressed SA‐β‐gal activity under matrix stiffening. Representative confocal images show networks stained for F‐actin (red), SA‐β‐gal (green), and nuclei (blue). For all immunofluorescence‐based analyses (M–P), each biological replicate (N = 3) included two independent hydrogel samples, with ≥ 3 fields of view per sample. Scale bars: 100 µm. Statistical analysis was performed using one‐way ANOVA followed by Tukey's multiple comparisons test. Data are presented as mean ± SEM. Significance levels were set at ns = not significant (*p* > 0.05), *p ≤ 0.05, **p ≤ 0.01, ***p ≤ 0.001, and ****p ≤ 0.0001.

To test this hypothesis, we examined the Notch receptor, ligands, and a downstream target gene across varying stiffness conditions. A previous study demonstrated stiffness‐dependent regulation of Notch signaling, with increased Notch activity and Dll4 expression on softer substrates [22]. In our 3D model, however, matrix stiffening elevated mRNA expression of *Notch1*, *JAG1, JAG2*, and the downstream target *HEY1*, with no significant change in *Dll4* ligand transcripts (Figure 3A). Protein analyses further confirmed increased NICD, JAG1, and JAG2 expression under matrix stiffening (Figure 3C–F), supporting activation of a Notch1‐JAG signaling under stiffened conditions. However, together with the selective induction of noncanonical alarmin‐ and stress‐associated secretory factors observed under matrix stiffening (Figure 2I–K), these findings suggest that additional stress‐responsive signaling pathways may also be engaged downstream of matrix stiffening [26].

Beyond its mechanosensitive role, Notch signaling has been linked to MAPK/JNK [27, 28] and NF‐κB [29] signaling under stress conditions. Since the stiffness‐induced secretory profile lacked canonical NF‐kB‐associated cytokines, including IL‐6, IL‐8, and CXCL1, we next examined JNK signaling and its downstream AP‐1 transcriptional program. Matrix stiffening significantly increased expression of *MAPK8* (encoding JNK1) and the AP‐1 components *FOS* and *JUN* (Figure 3B), consistent with activation of a JNK‐AP‐1‐associated stress‐response program alongside Notch signaling. At the protein level, phosphorylated JNK (p‐JNK) significantly increased in both cytoplasmic and nuclear fractions (Figure 3G–I). In contrast, phosphorylated c‐JUN (p‐c‐JUN) increased only in the cytoplasmic fraction, with no significant accumulation in the nucleus (Figure 3G,J,K). The observed JNK activation is consistent with a JNK‐AP‐1‐associated secretory remodeling program, as evidenced by the induction of MMP1 [30], MMP3 [30], and IL33 [31] under stiffened conditions.

To determine whether Notch signaling contributes to stiffness‐induced EC senescence, microvascular networks were pretreated with nirogacestat, an FDA‐approved γ‐secretase inhibitor that blocks NICD release and downstream Notch activation [32], for 3 h prior to matrix stiffening (Figure S3). Nirogacestat treatment reduced stiffness‐induced expression in *Notch1*, *MAPK8*, and *FOS* transcripts, whereas *JUN* expression remained elevated (Figure 3L). This suggests that Notch signaling contributes to the stiffness‐induced JNK/AP‐1 response, but does not fully account for it under our experimental conditions. Functionally, nirogacestat treatment reduced *CDKN1A* and *CDKN2A* gene expression (Figure 3L) and decreased both p16 staining intensity and SA‐β‐gal activity compared to untreated Stiff controls (Figure 3M–P), indicating attenuation of stiffness‐driven senescence. Together, these findings support a model in which matrix stiffening promotes endothelial senescence in part through a Notch‐associated stress‐response program.

### 2.4 Human Biospecimens Analysis Reveals Stiffness‐Induced Endothelial Senescence

To examine whether the stiffness‐associated endothelial senescence phenotype observed in our in vitro model is also present in humans, we analyzed fibrotic capsule specimens from patients with synthetic breast implants, which reproducibly develop localized fibrosis enriched with senescent cells [33, 34]. Oscillatory AFM‐based microrheology measurements showed increased relative stiffness in fibrotic capsule regions compared to adjacent non‐fibrotic tissues (Figure S5A).

**FIGURE 4:**
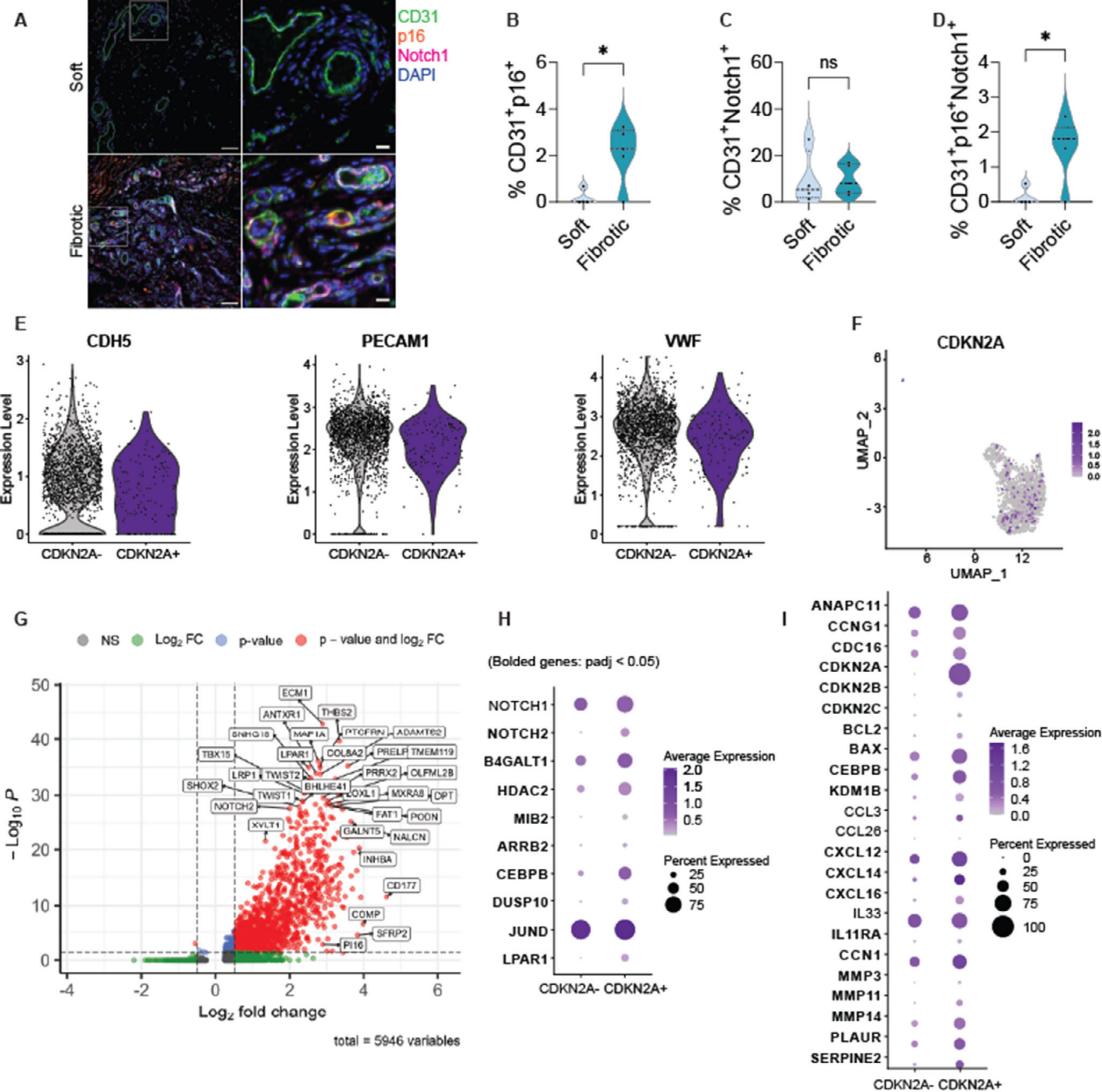
Human breast implant fibrotic capsule tissue confirms Notch‐driven endothelial senescence under stiffened environment. (A) Immunofluorescence staining of fibrotic capsules surrounding surgically explanted synthetic breast implants and adjacent fat tissue showed ECs (CD31) in green, senescence marker (p16^INK4a^) in orange, Notch1 receptor in magenta, and nuclei in blue. Representative images of both soft and fibrotic tissues were obtained from the same patient. Scale bar  =  100 µm (left), 20 µm (right). (B) Increased p16 expression was observed in ECs within fibrotic capsule regions compared to soft control tissues, including fat and muscle. (C) The proportion of non‐senescent ECs expressing Notch1 (CD31^+^Notch1^+^) was comparable between soft control and fibrotic tissues, suggesting Notch1 expression in non‐senescent ECs is not altered by stiffness. (D) A significantly higher frequency of triple‐positive (CD31^+^p16^+^Notch1^+^) cells in fibrotic regions indicates that Notch1 expression is enriched specifically within senescent ECs in stiffened environments. (E) Violin plots confirm EC identity through expression of standard EC markers VE‐Cadherin (CDH5), PECAM1, and von Willebrand factor (vWF). (F) UMAP projection identifies 132 *CDKN2A* ^+^ ECs out of 1836 total ECs. (G) Volcano plot showing differentially expressed genes between *CDKN2A* ^+^ and *CDKN2A* ^−^ ECs. Genes meeting adjusted *p* < 0.05 and log_2_ fold change > 0.5 are highlighted. (H) Dot plot displays enrichment of Notch‐ and JNK‐associated genes in senescent ECs. (I) *CDKN2A* ^+^ ECs show increased expression of genes related to cell cycle arrest (e.g., CDKN2A/B/C, CCNG1), SASP and inflammatory signaling (e.g., BCL2, BAX, CXCL12/14/16, IL11RA), and ECM remodeling (e.g., MMP3/11/14, PLAUR, SERPINE2, CCN1), supporting their active role in fibrosis‐associated tissue remodeling.

To assess EC senescence and Notch‐associated phenotypes in vivo, we performed triple immunofluorescence staining for CD31 (endothelial marker), p16 (senescence marker), and Notch1 (Notch signaling marker) in both fibrotic and adjacent soft control tissues (muscle and fat). The proportion of CD31^+^p16^+^ double‐positive cells was significantly higher in fibrotic capsules, indicating increased accumulation of senescent ECs in stiffened microenvironments (Figure 4A,B). While the percentage of CD31^+^Notch1^+^ double‐positive cells, representing Notch1 expression in non‐senescent ECs, remained similar between soft and fibrotic tissues (Figure 4C), the fraction of triple‐positive (CD31^+^p16^+^Notch1^+^) cells was significantly higher in fibrotic capsules (Figure 4D). This suggests that Notch1 receptor expression is preferentially increased in senescent ECs in vivo under fibrotic stiffening. Quantification of vessel area distribution showed no significant differences between soft control and fibrotic tissues (Figure S5C).

We next analyzed the endothelial subset from a previously published single‐cell RNA sequencing dataset of human fibrotic capsules surrounding synthetic breast implants [33]. Senescent ECs were identified based on expression of the cell cycle inhibitor *CDKN2A* (which encodes *16* ^Ink4a^), alongside standard EC markers, including *Cadherin‐5* (*VE‐C*ad), *PECAM‐1*, and *vWF* (Figure 4E) [35, 36]. Across the Uniform Manifold Approximation and Projection (UMAP) space, 132 of the total 1,836 ECs (∼7%) expressed p16 (Figure 4F), consistent with the expected low frequency of senescent cells in vivo [33]. This proportion is comparable to our immunofluorescence‐based quantification, which identified ∼12% of CD31^+^ cells as p16^+^, supporting the consistency of senescent EC detection across experimental modalities.

To confirm that *CDKN2A* ^+^ ECs represent a non‐proliferating population, we verified the absence of co‐expression with the proliferation marker MKI67. Given the known dropout rates of *CDKN2A* in single‐cell datasets, we further evaluated the robustness of our classification using multiple senescence gene signatures, including SenSig [33], SenMayo [37], CellAge [38, 39], and EC.SENESCENCE.SIG [40] (Figure S6A). *CDKN2A* ^+^ cells exhibited significantly higher module scores for SenSig, SenMayo, and CellAge compared to *CDKN2A* ^−^ cells, while EC.SENESCENCE.SIG was lower in *CDKN2A* ^+^ cells. When independently classifying senescent ECs based on the top 12% of signature scores, consistent with our immunofluorescence‐based prevalence, SenSig and SenMayo, most robustly enriched for cells expressing canonical senescence markers, including *CDKN2A* (p16) and *CDKN2B* (p15) (Figure S6B,C). Notably, 87% of differentially upregulated genes identified using *CDKN2A*‐based classification were also detected using SenSig and/or Sen Mayo scoring (Figure S6D). These results suggest that *CDKN2A*‐based classification captures similar senescent EC phenotypes as these signature‐based methods.

Differential gene expression analysis between *CDKN2A* ^+^ and *CDKN2A* ^−^ ECs identified 1487 genes with significant changes (adjusted *p* < 0.05). These genes were enriched in functional categories related to inflammatory signaling, ECM remodeling, and chromatin regulation (Figure 4G). While the volcano plot displays all genes detected in the analysis, only those meeting both statistical significance (adjusted *p* < 0.05) and a log_2_ fold change > 0.5 are highlighted as robustly regulated targets.

We next asked whether the transcriptomic features of *CDKN2A* ^+^ ECs were consistent with the pathway‐level changes observed in vitro. Analysis showed that *CDKN2A* ^+^ ECs expressed several key Notch pathway components (*Notch1/2, B4GALT1, HDAC2*, and *MIB2*) and JNK‐associated components (*ARRB2*, *CEBPB*, *DUSP10*, *JUND*, and *LPAR1*) (Figure 4H). Although Notch1 was expressed in a greater proportion of *CDKN2A* ^+^ cells, its expression level was comparable between the *CDKN2A* ^+^ and *CDKN2A* ^−^ groups.

Senescence‐associated genes were further profiled (Figure 4I). Genes involved in cell cycle regulation (*ANAPC11*, *CCNG1*, *CDC16*, *CDKN2A*, *CDKN2B*, and *CDKN2C*) were differentially expressed in *CDKN2A* ^+^ cells, consistent with a senescent phenotype. SASP‐related genes, including *BCL2* and *BAX* genes, as well as chromatin regulators such as *CEBPB* and *KDM1B*, were also enriched in the *CDKN2A* ^+^ population. Moreover, immune signaling molecules such as *CXCL12*, *CXCL14*, *CXCL16*, and *IL11RA* showed elevated expression, suggesting an immunomodulatory and pro‐inflammatory profile in senescent ECs. Importantly, genes identified from our in vitro Luminex analysis, including *CCL3*, *CCL26*, and *IL33*, were expressed by a higher proportion of *CDKN2A* ^+^ cells, even though their average expression levels remained similar between groups.

Lastly, ECM remodeling genes such as *CCN1*, *MMP3*, *MMP11*, *MMP14*, *PLAUR*, and *SERPINE2* were significantly upregulated in the *CDKN2A* ^+^ group (Figure 4I). Gene Set Enrichment Analysis (GSEA) further demonstrated enrichment of pathways related to collagen fibril organization and collagen metabolism in the senescent EC population (Figure S7). Furthermore, ECM organization‐related pathways were consistently enriched across all classification methods (Figure S7), indicating that this ECM‐associated signature is robust across classification strategies.

## 3 Discussion

This study demonstrates that dynamic matrix stiffening induces senescence in lumenized endothelial microvascular networks and reshapes the endothelial senescence‐associated responses. We developed a tunable 3D hydrogel platform that enables the formation of microvascular networks within a soft, collagen‐rich matrix, followed by controlled, on‐demand stiffening in the absence of exogenous inflammatory stimulation. This system enabled direct assessment of how dynamic mechanical transitions influence EC network senescence in a 3D context. Consistent with previous studies linking matrix stiffness to senescence in vascular smooth muscle cells [41], alveolar epithelial cells [42], and myofibroblasts [43], our findings extend this concept to endothelial networks and show that matrix stiffening alone is sufficient to induce hallmark senescence‐associated phenotypes in ECs. Importantly, these effects were not attributable to radical exposure, as all stiffened groups were subjected to identical photopolymerization parameters and exhibited a gradual increase in senescence marker expression with increasing stiffness.

While matrix stiffness was the primary biomechanical cue examined in the present study, ECM viscoelasticity and plasticity are additional matrix properties that can independently regulate mechanotransduction and endothelial fate [44, 45, 46]. As native ECMs are viscoelastic, future versions of this platform could incorporate these features to better capture physiologic matrix mechanics and to determine whether they further modulate endothelial senescence.

In classical forms of senescence induced by replication stress or DNA damage, the SASP is typically regulated by NF‐κB‐associated pro‐inflammatory cytokine secretion, including IL‐6, IL‐8, and CXCL1 [47, 48, 49]. In contrast, the SASP induced by matrix stiffening exhibited a distinct immunomodulatory profile, with selective upregulation of IL‐33, IL‐1α, and IFN‐γ, factors associated with alarmin signaling and stress‐responsive inflammatory regulation [50, 51, 52]. Together with suppression of canonical NF‐κB‐associated cytokines, these findings suggest that mechanical stress may shift endothelial senescence toward a non‐canonical secretory state rather than a broad inflammatory SASP program. Such a phenotype may be particularly relevant in localized fibrotic microenvironments, where senescent ECs may reshape the behavior of adjacent stromal and immune cells through paracrine signaling [53].

To investigate signaling pathways associated with this response, we focused on Notch signaling due to its established roles in endothelial mechanosensing [22], vascular homeostasis [21], and vascular cell senescence [24]. Previous studies have shown that Notch signaling exhibits both protective and pathological effects in the vasculature, depending on the microenvironmental context and receptor‐ligand pairing [24, 25, 54, 55]. While previous studies reported enhanced Notch1‐Dll4 signaling on softer substrates [22], our 3D model demonstrated that matrix stiffening upregulates Notch1 and JAG ligands at both the transcript and protein levels, supporting activation of a Notch1–JAG signaling under stiffened conditions. Notably, the Notch1‐JAG axis has been implicated in pathological vascular remodeling and inflammatory responses [56], suggesting that targeting Notch1 activation could offer an effective therapeutic strategy for stiffness‐induced endothelial dysfunction.

Mechanistically, our findings support coordinated activation of Notch signaling and a stress‐responsive JNK‐AP‐1 program under matrix stiffening. Notch inhibition attenuated stiffness‐induced senescence responses, as reflected by reduced p16 expression and SA‐β‐gal activity, indicating that Notch signaling plays a functional role in this process. Given that previous studies have shown that loss of JAG ligands can enhance complementary Notch1–Dll4 signaling [57], use of a pan‐Notch inhibitor in our model enabled broader assessment of the Notch pathway involvement rather than interrogation of individual receptor‐ or ligand‐specific contributions. Future studies using more targeted genetic approaches, such as Notch1 knockdown or receptor‐specific perturbation strategies, will help clarify the relative contributions of individual Notch components in stiffness‐induced endothelial senescence.

As a demonstration of the physiological relevance of our in vitro findings, we extended our validation to a human tissue model using fibrotic capsules from patients with breast implants. Unlike chronic fibrotic diseases characterized by prolonged systemic inflammation and complex immune pathologies, this foreign body response model reflects localized fibrotic remodeling driven predominantly by mechanical forces and the local microenvironment [58]. Previous studies have shown that fibrotic capsules from human breast implants exhibit 1–30 MPa elastic moduli [59, 60, 61], whereas adjacent skeletal muscle (10–100 kPa) [62, 63, 64] and adipose tissues (0.5–30 kPa) [65] are orders of magnitude softer, demonstrating the substantial mechanical changes associated with fibrotic remodeling. Given that our in vitro hydrogel platform recapitulates a mechanically induced EC senescence program without chronic, systemic inflammatory confounders, we leveraged this model as a relevant in vivo system to study mechanically driven fibrosis and senescence.

Immunofluorescence and scRNA‐seq analyses confirmed the presence of a stiffness‐responsive, senescent endothelial population in fibrotic capsule regions. These cells exhibited enrichment of Notch and JNK signaling components, along with inflammatory and ECM remodeling genes. In contrast, senescent ECs in our short‐term in vitro system exhibited a more limited fibrotic interstitial remodeling signature. This discrepancy likely reflects differences in cellular composition and time scale between the in vitro system and human fibrotic tissue context, where paracrine signaling and longer‐term feedback interactions may further amplify ECM remodeling responses. These findings suggest a potentially bidirectional relationship in which matrix stiffening promotes endothelial senescence, while senescent ECs may subsequently contribute to progressive matrix remodeling through paracrine signaling and sustained tissue remodeling [66, 67].

Nonetheless, several considerations should be noted. While this foreign body response model offers a localized fibrosis environment focused on mechanical remodeling, physiological vascular aging also involves systemic factors [68], such as chronic inflammation, metabolic dysregulation, and cumulative hemodynamic stress, which are not fully captured here. In addition, the relatively low number of senescent ECs identified in this dataset (∼132 cells) limited our ability to resolve heterogeneity within this population or perform robust differential expression analysis across senescent EC subpopulations. Larger datasets enriched for senescent ECs will be important in future studies to define the diversity and functional states of senescent endothelial populations.

Overall, this work identifies matrix stiffening as a driver of endothelial senescence and supports a mechanosensitive Notch‐associated program linked to stress‐associated secretory and remodeling responses. These findings further support a bidirectional relationship between matrix stiffening and endothelial senescence and highlight potential therapeutic interventions for vascular aging and fibrotic disease.

## 4 Experimental Methods

### 4.1 Synthesis of Methacrylated HA (HA–MA)

HA‐MA was synthesized as previously reported [69]. Approximately 14 or 64% of the primary hydroxyl groups were modified with methacrylate groups. Briefly, 2% (w/v) solution of HA (Lifecore, 60 kDa) was prepared on ice and reacted with methacrylic anhydride (Sigma) at HA/MA molar ratios of 1: 0.5 and 1:3 to achieve ∼14% and ∼64% modification, respectively. The pH was maintained at 8–9 for 4 h. The products were dialyzed against NaCl Solution for 48 h, lyophilized, and the degree of modification was determined with ^1^H NMR (Bruker).

### 4.2 Formation and Characterization of Hydrogels

Stock solutions of Col‐MA (8 mg/mL, Advanced Biomatrix) and HA‐MA (20 mg/mL in PBS) were prepared separately. To initiate collagen physical cross‐linking, a defined volume of neutralization solution (Advanced Biomatrix) was added to the Col‐MA solution to adjust pH. The pH‐adjusted solution was then mixed with HA‐MA solution and PBS to achieve a final concentration of 2.5 mg/mL Col‐MA and 0.25 mg/mL HA‐MA. The resulting precursor mixture was incubated at 37°C for 30 min to form a physically cross‐linked Col‐HA hydrogel. Following gelation, the photoinitiator was prepared according to the manufacturer's instruction (Advanced Biomatrix). Briefly, Ruthenium (Ru, 28 mg/mL) and Sodium Persulfate (SPS, 119 mg/mL) were diluted in PBS to final concentrations of 0.7 and 2.4 mg/mL, respectively. This solution was added on top of the gels and incubated for 10 min to allow diffusion into the gels. To induce secondary chemical cross‐linking and stiffening, hydrogels were then exposed to white LED light for 1 min.

Hydrogel bulk stiffness was characterized using a Discovery Hybrid Rheometer (HR20, TA Instruments) equipped with an 8 mm cross‐hatched geometry and a 500 um gap, maintained at 37°C. Premade hydrogel disks (140 µL, 8 mm diameter) were placed on the rheometer stage, and PBS was added around the geometry to ensure the hydrogels remained swollen. An amplitude sweep was performed from 0.01% to 10% strain at a constant frequency of 0.25 Hz. Storage modulus (G’) values were recorded at 0.1% strain within the linear viscoelastic regime. Young's modulus (E) was calculated from the shear modulus using the equation: E = 2G (1 + ν) (E is Young's modulus, G is shear modulus, and ν is Poisson's ratio), assuming a Poisson ratio of 0.5 [70].

Microscale mechanical properties of hydrogels and human breast implant fibrotic capsule tissues were characterized by atomic force microscopy (AFM)‐based microrheology [12] using a Bruker NanoWizard V instrument. Hydrogel samples were prepared by placing them onto glass coverslips, with all measurements performed while samples were submerged in PBS prior to and during data acquisition. Force spectroscopy measurements were carried out using SAA‐SPH‐1UM cantilevers (nominal spring constant ∼0.15 N/m). For hydrogel characterization, microrheology curves were acquired at a frequency of 1 Hz, with a normal force of 100 pN, an approach distance of 10 µm, and an approach velocity of 2 µm/s. For each sample, measurements were collected over a 5 × 5 grid spanning an area of 10 × 10 µm. A total of three biological replicates (independently prepared hydrogel samples) were analyzed. Curves were processed using the Bruker Data Processing software. Curves were filtered by fit. For human breast implant fibrotic capsule tissue, AFM‐based microrheology measurements were performed under similar conditions, except using a 20 nm amplitude/strain, a 5 µm approach distance, and a normal force of 3 nN at a frequency of 1 Hz. Measurements were acquired across multiple locations in each tissue region to account for tissue heterogeneity. Because measurements were performed on fixed tissue samples, the resulting values were interpreted as relative mechanical properties.

To assess the equilibrium swelling ratio, premade hydrogels were formed in pre‐weighted cell culture insert wells. After full swelling, the weight of the hydrated hydrogels was measured. The dry hydrogel weight was obtained by incubating the hydrogels at 60°C until completely dehydrated. The swelling ratio (%) was calculated as:

$$Swelling\;\;ratio\;\;\left(\%\right)=\left(\frac{Swollen\;weight-Dry\;weight}{Dry\;weight}\right)\times 100$$

Collagen‐HA fibers were imaged using reflective microscopy (640 nm, Nikon Eclipse Ti2 microscope). Fiber diameter and coverage area were quantified using Nikon NIS software. A total of 25 images acquired from 4 replicates were analyzed for quantification.

### 4.3 Cell Culture

All cells were maintained in a humidified incubator at 37°C with 5% CO_2_. Human umbilical cord blood‐derived ECFCs (provided by the Yoder Lab, Indiana University School of Medicine) were selected for their well‐established proliferative and vasculogenic potential, including their ability to form lumenized microvascular networks in 3D hydrogel environments [71]. ECFCs were cultured in EGM2 (Lonza) supplemented with an additional 10% HyClone FBS, beyond the standard formulation. For 2D expansion, cells were seeded on type I collagen (Corning)‐coated tissue culture plates. For 3D hydrogel cultures, the media were further supplemented with 50 ng/mL of VEGF (PeproTech) and 25 ng/mL of bFGF (PeproTech) to support endothelial network formation.

### 4.4 3D Cell Encapsulation and Dynamic Matrix Stiffening

The hydrogel precursor mixture was prepared and neutralized as described above. ECFCs were suspended in EGM2 supplemented with 50 ng/mL VEGF and 25 ng/mL bFGF and mixed with the hydrogel solution to achieve a final cell concentration of 2 million/mL. Cell‐laden hydrogels were initially cultured under soft matrix conditions for 48 h to allow the formation of lumenized endothelial networks. On Day 2, photoinitiator solution was added to all hydrogel conditions, and dynamic matrix stiffening was induced in the IM and Stiff groups by 1‐minute white LED exposure, as described above, while Soft controls remained unstiffened. Following stiffening, constructs were maintained for an additional 48 h prior to endpoint analyses.

### 4.5 Hydrogel Mass Stability Assessment

To evaluate hydrogel stability during the post‐stiffening culture period, the wet mass of cell‐laden hydrogels was measured on Days 2, 3, and 4 following photo‐stiffening. Hydrogels were maintained under standard culture conditions, and mass measurements were collected every 24 h. Relative mass change was calculated by normalizing to the Day 2 wet mass.

### 4.6 Cell Viability

Cell viability was analyzed by Live/Dead staining using calcein‐AM, ethidium homodimer, and Hoechst 33342 on Day 4. Cell viability was quantified using ImageJ by counting the total number of cells (stained by Hoechst) and dead (stained by ethidium homodimer) cells in each image of maximum intensity projection produced by Nikon NIS analysis software.

### 4.7 Hydrogel Immunofluorescent Staining, Imaging, and Quantification

To enable reliable assessment of p16 expression in the 3D hydrogel system, immunofluorescence‐based analysis was used, allowing single‐cell level visualization in the intact matrix. On day 4, cell‐laden hydrogel constructs were fixed with 4% (w/v) paraformaldehyde in PBS for 45 min and then blocked/permeabilized with 5% (w/v) bovine serum albumin and 0.1% (v/v) Triton X‐100 for 4 h at room temperature. The constructs were incubated with primary antibodies, including anti rabbit‐p16 (1:400, Proteintech) and anti mouse‐VE‐CAD (1:400, Santa Cruz), overnight at 4°C. Subsequently, the constructs were incubated with corresponding secondary antibodies or phalloidin (Invitrogen). Nuclei were stained with DAPI. Fluorescent images were taken by Nikon EclipseTi2 microscope using Z‐stack mode. Maximum intensity projection images were generated by Nikon NIS analysis software. Image quantification was conducted using ImageJ and Imaris.

SA‐β‐Gal staining was performed using CellEvent Senescence Green Detection Kit (Thermo Fisher) according to the manufacturer's instructions with minor modifications. Day 4 cell‐laden hydrogel constructs were fixed and then blocked/permeabilized with 1% (w/v) bovine serum albumin and 0.1% (v/v) Triton X‐100 for 30 min at room temperature. The constructs were subsequently incubated with prewarmed working solution for 3 h at 37°C without CO_2_ and protected from light. Phalloidin (Invitrogen) and DAPI were used for counterstaining. Imaging and quantification were performed as described above.

### 4.8 Luminex Analysis

Custom Luminex assay kits (Bio‐Techne) were used to quantify soluble factors. Cell culture supernatants were collected on day 4 of culture from hydrogels under Soft, IM, and Stiff conditions (N = 2, n = 5). Fresh cell culture media served as the blank control. All Luminex assays were performed in the Regional Biocontainment Laboratory (RBL) at Duke University, following the manufacturers’ instructions. Analyte concentrations were normalized to dsDNA content, measured using the PicoGreen assay (Invitrogen, N = 2, n = 7), following previously published procedures [72].

### 4.9 Small‐Molecule Inhibition Studies

Nirogacestat, a γ‐secretase inhibitor, was used to inhibit Notch receptor cleavage and downstream Notch activation. A 10 mM stock solution of nirogacestat in DMSO was diluted to a final concentration of 750 nM in EGM2 supplemented with 50 ng/mL VEGF and 25 ng/mL bFGF. Cell‐laden hydrogel constructs were pretreated with nirogacestat‐containing media for 3 h prior to matrix stiffening on Day 2 post‐encapsulation. Following light‐induced stiffening, constructs were continuously maintained in nirogacestat‐containing media throughout the subsequent 48‐h culture period until Day 4 endpoint analysis.

### 4.10 Gene Expression

After 4 days of culture, cell‐laden constructs were flash‐frozen in liquid nitrogen and stored at −80°C until further processing. Frozen samples were thoroughly homogenized in TRIzol (Invitrogen) and extracted RNA was purified using the RNA Clean & Concentrator‐5 Kit (Zymo Research) according to the manufacturer's protocol. Complementary DNA was synthesized using GoScript Reverse Transcription Mix (Promega). Quantitative real‐time PCR was performed using Maxima SYBR Green/Fluorescein Master Mix (ThermoFisher). Primer sequences are listed in Table S4. The *MAPK8*, *FOS, CYR61, CTGF, and MMP9* primers were purchased from Millipore Sigma. GAPDH was used as a reference gene. The fold change was processed by qbase+ software (Biogazelle). A total of three biological repeats were analyzed for each gel composition.

### 4.11 Western Blotting

Day 4 frozen constructs were lysed in RIPA buffer (Thermo Fisher) containing 1% (v/v) of Halt protease and phosphatase inhibitor cocktail (Thermo Fisher). For nuclear and cytoplasmic protein separation, the NE‐PER Nuclear and Cytoplasmic Extraction Reagent kit was used following the manufacturer's instructions. Protein concentrations were determined using the BCA assay kit (Thermo Fisher). Samples were denatured by boiling at 95°C for 10 min, and 20–35 µg of protein was loaded per lane on 4%–20% or 7.5% precast polyacrylamide gel (Bio‐Rad). Proteins were transferred to Immun‐Blot PVDF Membrane (Bio‐Rad) overnight at 4°C. Protein transfer was confirmed by Ponceau‐S staining. Membranes were blocked for 1 h with 5% BSA in TBST for the detection of phosphorylated proteins, and 5% milk in TBST for non‐phosphorylated proteins. Membranes were then incubated overnight at 4°C with primary antibodies targeting: p21 (Cell Signaling, rabbit, 1:1000), NICD (Cell Signaling, rabbit, 1:1000), JAG1 (Cell Signaling, rabbit, 1:1000), JAG2 (Cell Signaling, rabbit, 1:1000), phospho‐JNK (Cell Signaling, rabbit, 1:750), JNK (Cell Signaling, rabbit, 1:750), phospho‐c‐JUN (Cell Signaling, rabbit, 1:750), c‐JUN (Cell Signaling, rabbit, 1:750), and PCNA (Proteintech, rabbit, 1:1000). Following primary antibody incubation, blots were washed three times with TBST and incubated with species‐appropriate HRP‐conjugated secondary antibodies or HRP‐conjugated GAPDH (Cell Signaling, rabbit, 1:2500). Immunoblots were detected by an enhanced chemiluminescence western blotting substrate (Thermo Fisher) and imaged using ChemiDoc Imaging System (Bio‐Rad). Quantification was performed using Bio‐Rad Image Lab software. Phosphorylated protein levels were normalized to their total protein counterparts. Original blots were included in Figure S8.

### 4.12 Human Tissue Samples

Deidentified surgical discards from patients undergoing breast implant exchange or replacement surgeries were collected under Johns Hopkins University Institutional Review Board Exemption IRB00088842. For single‐cell RNA‐sequencing, cells were isolated from the fibrotic capsules from one patient, as previously published [33]. For immunofluorescence staining and imaging, discarded fibrotic capsule tissue and implant‐adjacent muscle and fat tissue were obtained from six patients. All tissues were immediately fixed in 10% formalin for 48 h. Tissues were then rinsed twice in PBS, dehydrated using a graded ethanol series (70%, 80%, 95%, 100%, 100% ethanol for 1 h each), and cleared in xylene for 1–2 h. Tissues were incubated in 4–6 exchanges of melted paraffin wax at 60°C for 24 h to prepare for embedding. Tissues were embedded in wax blocks using metal molds and stored at room temperature until sectioning. Embedded tissues were sectioned at 5 µm using a microtome.

### 4.13 Single Cell RNA Sequencing

Single‐cell RNA‐sequencing data were obtained from a previously published dataset of cells isolated from human breast implant fibrotic capsule tissues [33]. The processed scRNA‐seq dataset of the human breast implant fibrotic capsule is available for download on Zenodo using the following DOI: https://doi.org/10.5281/zenodo.20017381. All analyses were performed using the Seurat v5.1 package [73]. Endothelial cells were identified based on their expression of known markers (PECAM1, CDH5, VWF) and were subsetted for downstream analysis. Cells that were positive for CDKN2A (p16) expression were annotated as senescent. All p16^+^ cells were negative for the proliferation marker MKI67. Differential expression analysis was performed between senescent (p16^+^) and non‐senescent (p16^−^) cells using a Wilcoxon Rank Sum test via the FindMarkers function in Seurat, with thresholds of logFC ≥ 0.25 and min.pct = 0.1. Genes with an adjusted p‐value below 0.05 were considered differentially expressed. Differentially expressed genes were visualized using the Seurat, EnhancedVolcano v1.22.0 [74], and ggplot2 packages. Gene set enrichment analysis (GSEA) was performed using the FindMarkers function in Seurat, followed by the Fast Gene Set Enrichment Analysis (fgsea) v1.30.0 package [75]. Genes were ranked for GSEA analysis using two steps. First, differential expression was performed using the FindMarkers function with thresholds of logFC ≥ 0, min.pct = 0, minimum.cells.group = 1, and min.cells.feature = 1. Then, genes were ranked in descending order by their values of the following calculation: −log10(*p*‐value) * avg_log2FC. Ranked genes were input into fgsea to identify pathways enriched in senescent endothelial cells. GSEA was performed using the human HALLMARK, REACTOME, KEGG, and GOPB databases. Pathways with a false discovery rate of <0.05 were considered significant.

Senescence signature scoring was performed using the AddModuleScore function in Seurat v.5.4 [73]. The following publicly available signatures were used for senescence scoring: SenSig [33], SenMayo [37], CellAge [38, 39], and EC.SENESCENCE.SIG [40]. For SenSig, the genes were filtered prior to analysis to include only those with a positive logFC and FDR < 0.05. Cells within the top 12% of each signature were classified as senescent. For each senescence scoring method, differential expression analysis and GSEA between senescent and non‐senescent ECs were performed as described in the previous paragraph.

### 4.14 Immunofluorescence Staining of Human Tissues

Tissue sections were stained using the PhenoCode Signature platform by Akoya Biosciences. Slides were incubated in an oven at 60°C to remove excess paraffin wax. Then, slides were deparaffinized in xylenes for 3 × 3 min and rehydrated in a graded series of ethanol (100% x 2, 95%, 80%, 70%; 1 min each) and Millipore water (3 × 3 min). Antigen retrieval was performed by incubating slides in pre‐heated 1X AR6 buffer in a vegetable steamer for 15 min. Slides were cooled at RT for 15 min, rinsed in Millipore water, and incubated in 3% H_2_O_2_ for 15 min to block endogenous peroxidases. Then, slides were rinsed once in Millipore water and incubated in a blocking buffer of 10% bovine serum albumin and 0.05% Tween‐20 in 1X PBS for 30 min at RT. Slides were stained with the following antibodies in blocking buffer at RT: p16 for 15 min (CINTEC‐Roche, 705–4793, 1:5), Notch1 for 30 min (Cell Signaling, 3608, 1:200), and CD31 for 30 min (Abcam, ab76533, 1:100). Following the primary antibody incubation, slides were washed in 1X TBST for 3 × 3 min and incubated in secondary antibody for 30 min at RT. We used MACH 4 Universal HRP‐Polymer (Biocare Medical, M4U534) to detect p16 and MACH 3 Rabbit HRP Polymer Detection (Biocare Medical, M3R531) to detect Notch1 and CD31. After TBST washes, slides were incubated in Opal dyes diluted in 1X Manual Amplification Diluent for 10 min at RT. Opal dyes 570, 650, and 520 were used to label p16, Notch1, and CD31, respectively. Slides were washed in TBST for 3 × 3 min. Incubations with AR6 buffer, blocking buffer, primary antibodies, secondary antibodies, and Opal dyes were repeated three times to enable co‐staining of p16, Notch1, and CD31. Only one antibody was used for staining in each round. Following the three rounds of antibody staining, slides were rinsed in Millipore water, stained with 1X Spectral DAPI solution for 5 min, rinsed twice in Millipore water, and mounted in DAKO Fluorescence Mounting Medium (Agilent, S302380‐2) using #1.5 coverslips. Slides were dried overnight and stored at 4°C until imaging. Slides were imaged using the Zeiss Axio Observer.Z1/7 with Apotome and processed using ZenBlue software.

### 4.15 Colocalization and Vessel Area Analysis of Human Tissues

Images of human breast implant tissues were analyzed for colocalization of senescence and vascular markers (CD31, Notch1, and p16) using HALO v3.6 with the HighPlex FL v3.2 module (Indica Labs). Regions of interest (ROIs) were drawn to select the regions with the fibrotic capsules (stiff) and muscle or fat (soft) in each image. Nuclear detection was performed using the DAPI stain and cytoplasmic detection was used for the antibody stains. Minimum intensity thresholds were set for each stain and were kept consistent across images. Primary delete slides for each antibody stain were used as negative controls to minimize false‐positive signal detection. Colocalization of the antibody stains in each DAPI^+^ cell was measured, exported, and analyzed in GraphPad Prism 10.

Vessel area and density measurements were performed using a custom script in QuPath 0.5.1. ROIs were drawn using the same approach as in the colocalization analysis. Vessel detection was performed using the OpenCVPixelClassifer function. A gaussian blur and minimum intensity threshold were applied to detect areas positive for CD31 (vessel marker). Vessel areas <20 µm^2^ or >900 000 000 µm^2^ were excluded from the analysis. The resulting vessel annotation in each image was split to enable the analysis of individual vessel fragments. Tissue ROI and vessel area measurements were exported and analyzed using GraphPad Prism 10.

### 4.16 Statistical Analysis

The authors performed statistical analysis using GraphPad Prism 10 (GraphPad Software Inc.). This software was also used to perform t‐tests, one‐way ANOVA, and two‐way ANOVA to determine significance. Replicates were indicated throughout the figure captions. Significance levels were set at *p < 0.05, **p < 0.01, ***p < 0.001, and ****p < 0.0001.

## Author Contributions

**Jiyeon Song**: conceptualization, investigation, writing – original draft, methodology, visualization, writing – review and editing, formal analysis, data curation. **Alexandra N. Rindone**: investigation, writing – review and editing, data curation. **Ya Guan**: methodology, writing – review and editing. **Connor D. Amelung**: investigation, methodology, formal analysis. **Prarthana Sanjay Daswani**: formal analysis. **Jennifer H. Elisseeff**: supervision, resources, methodology, writing – review and editing, funding acquisition. **Sharon Gerecht**: conceptualization, funding acquisition, writing – original draft, writing – review and editing, supervision, resources.

## Conflicts of Interest

The authors declare no conflicts of interest.

## Supporting information




**Supporting File**: advs76304‐sup‐0001‐SuppMat.pdf.

## Data Availability

The data supporting the findings of this study are publicly available in the Duke University Libraries Research Data Repository (https://doi.org/10.7924/r4r535). The processed human breast implant fibrotic capsule scRNA‐seq dataset is available on Zenodo (https://doi.org/10.5281/zenodo.20017381). Additional human tissue data are available on Zenodo (https://zenodo.org/records/20804465).
