# Supplementary material for: Matrix Stiffness Induces Endothelial Network Senescence
Source: Adv Sci (Weinh). 2026 Jul 2:e76304. Online ahead of print. doi: 10.1002/advs.76304 (PMC13337105; doi:10.1002/advs.76304)
Supplement: Supplementary file 1 — Supporting File: advs76304‐sup‐0001‐SuppMat.pdf. [file ADVS-9999-e76304-s001.pdf]

Matrix stiffness induces endothelial network senescence

Jiyeon Song[1], Alexandra N. Rindone[2], Ya Guan[1], Connor D. Amelung, Prarthana Sanjay Daswani[2], Jennifer H. Elisseeff[2,3,4], and Sharon Gerecht[1]

Supplementary Materials

Supplementary Figures:

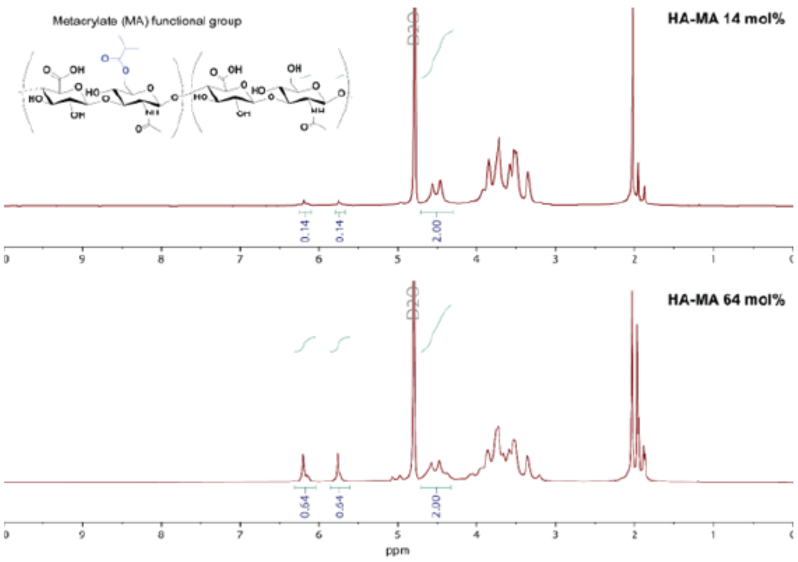

Fig. S1. 1H NMR spectra for HA-MA low (14 mol%, top) and high (64 mol%, bottom) in D_2O

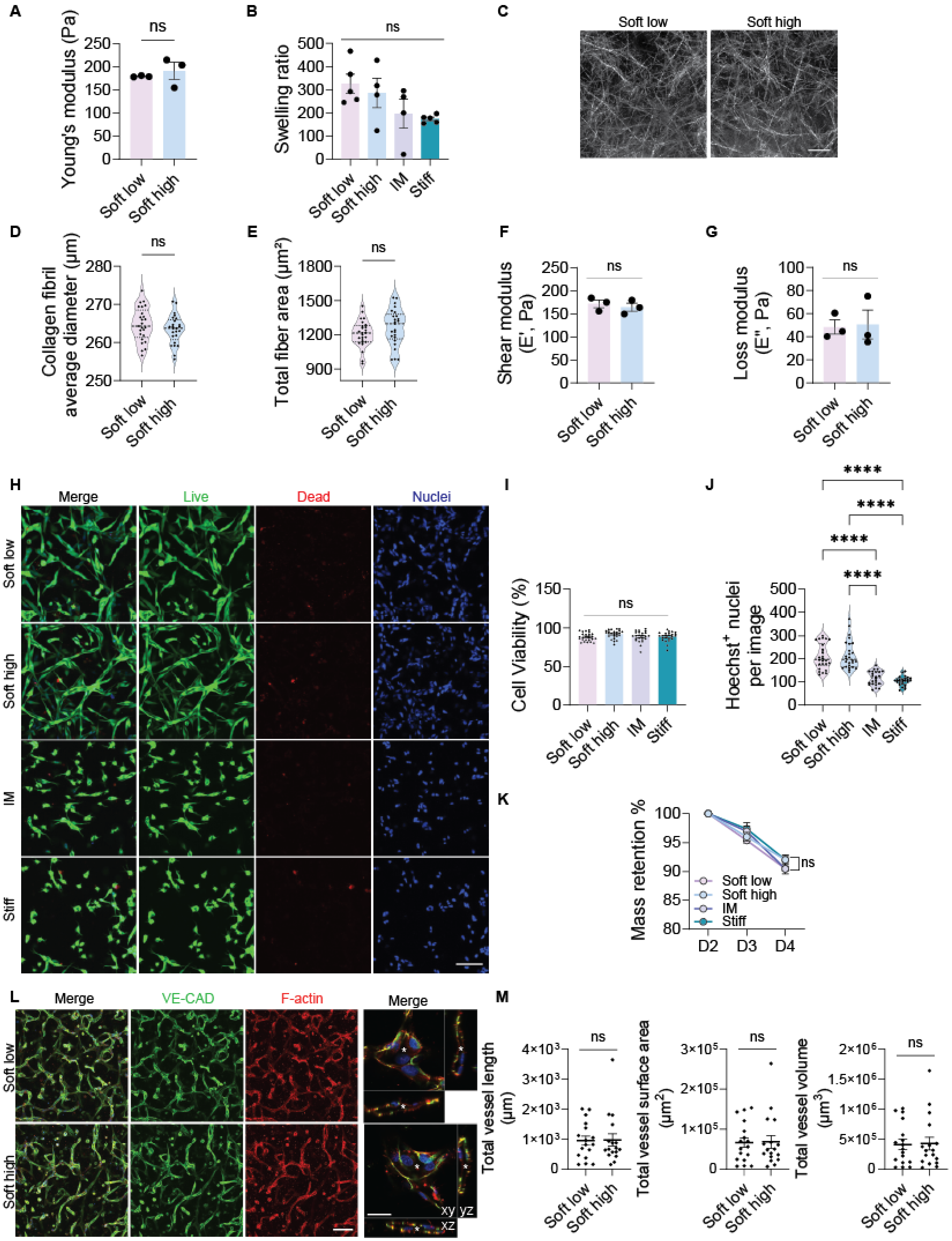

A
Young's modulus (Pa)
ns
Soft low
Soft high
B
Swelling ratio
ns
Soft low
Soft high
IM
Stiff
C
Soft low
Soft high
D
Collagen fibril average diameter (μm)
ns
E
Total fiber area (μm²)
ns
F
Shear modulus (E', Pa)
ns
G
Loss modulus (E'', Pa)
ns
H
Merge
Live
Dead
Nuclei
Soft low
Soft high
IM
Stiff
I
Cell Viability (%)
ns
J
Hoechst+ nuclei per image
****
K
Mass retention %
ns
D2
D3
D4
L
Merge
VE-CAD
F-actin
xy
yz
xz
M
Total vessel length (μm)
Total vessel surface area (μm²)
Total vessel volume (μm³)
ns

Fig. S2. Hydrogel characterization of formulations with varying methacrylate incorporation on HA, including two Soft formulations. (A) Young's modulus measured by rheometry. (B) Equilibrium swelling ratio. (C) Representative confocal reflectance images showing comparable fibrillar microstructure (scale bar: 20 μm) as quantified in (D) by collagen fibril average diameter and in (E) by total fiber area. N=5 with 5 fields of images in each. (F and G) Microscale viscoelastic characterization measured by AFM-based microrheology: Shear modulus in (F) and loss modulus in (G). (H) Representative images of Live/Dead staining with Hoechst nuclear staining of ECs cultured in Soft low, Soft high, Intermediate (IM), and Stiff 3D on-demand stiffening hydrogels at Day 4. Live cells are shown in green (calcein AM), dead cells in red (ethidium homodimer), and nuclei in blue (Hoechst 33342). Each biological replicate (N=3) included two independent hydrogel samples, with ≥4 fields of view per sample analyzed. Scale bar = 100 μm. (I) Quantification of cell viability, presented as the percentage of live cells relative to total cells. (J) Quantification of total cell number per field of view across hydrogel conditions, derived from the Live/Dead datasets used in (H) and (I). Each data point represents the total cell count per field of view. (K) Quantification of hydrogel wet mass over 48 hours following D2 stiffening. Cell-laden hydrogels exhibited ~10% mass loss with no significant differences between conditions (N=2 biological replicates with three independent hydrogels per condition). (L) Representative maximum intensity projections of confocal z-stack showing microvascular networks and orthogonal cross-sections of 3D lumina (asterisks) in Soft low and Soft high hydrogels. Scale bars: 100 μm (networks) and 25 μm (lumina). (M) Quantification showing comparable microvascular network formation in terms of total vessel length, surface area, and volume. Each biological replicate (N=3) included two independent hydrogel samples, with ≥2 fields of view per sample analyzed. Scale bar = 100 μm. Statistical analyses were performed using Student's t-test or one- or two-way ANOVA followed by Tukey's multiple comparisons test, as appropriate. Data are presented as mean ± SEM. Significance levels were set at ****$p \leq 0.0001$.

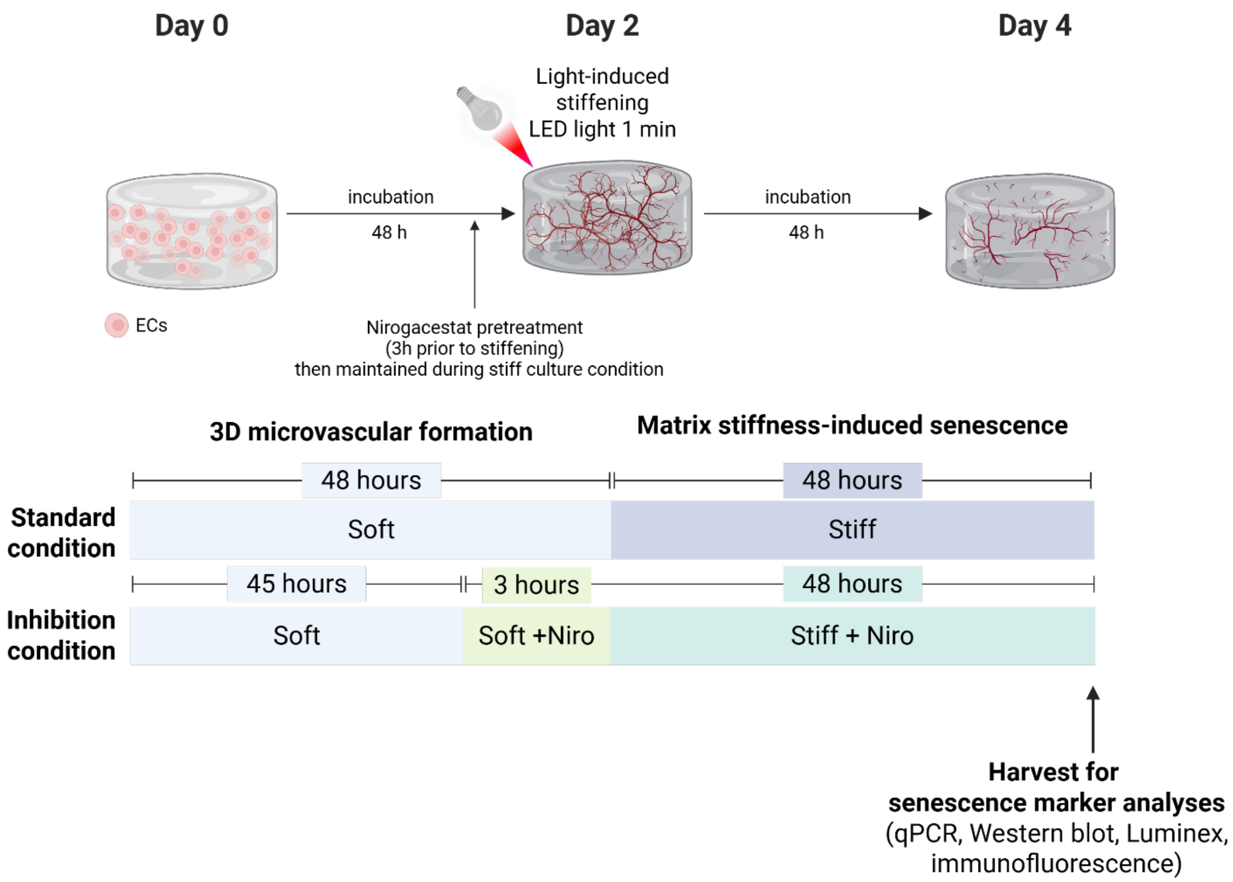

Fig. S3. On-demand hydrogel stiffening system and experimental timeline. Lumenized EC networks were formed under soft matrix conditions. On Day 2, photoinitiator solution was added to all hydrogel conditions. IM and Stiff conditions were then generated via 1-minute white LED-induced hydrogel stiffening, while Soft controls remained unstiffened. Following stiffening, EC networks were cultured for an additional 48 hours with media changes every 24 hours. On Day 4, hydrogel samples were collected for immunofluorescence, qPCR, Western blotting, and Luminex analysis of culture supernatants. For Notch inhibition studies, EC networks were pretreated with nirogacestat for 3 hours prior to stiffening and maintained in nirogacestat-containing media throughout the subsequent 48-hour culture period.

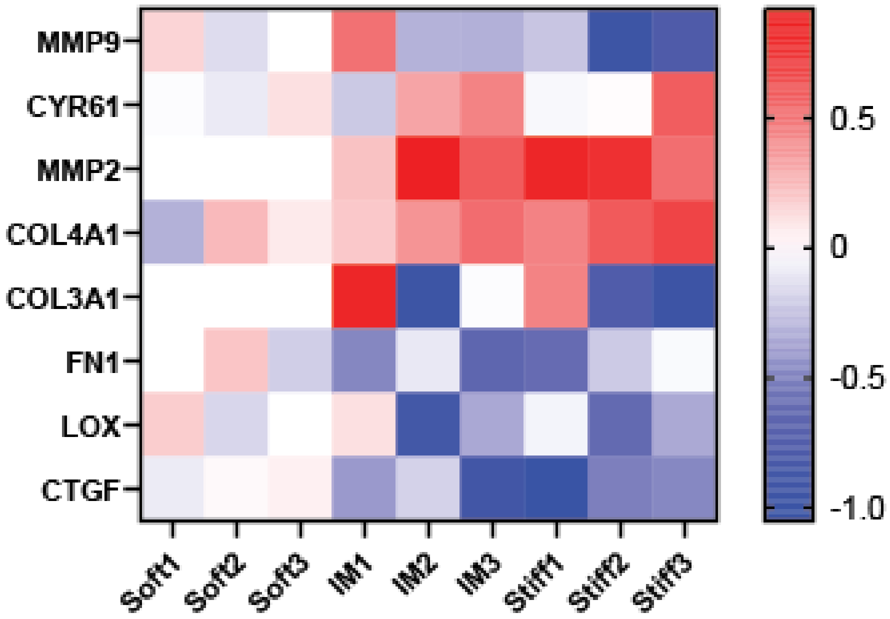

Fig. S4. Matrix stiffening alters angiogenesis- and ECM remodeling-associated gene expression in endothelial microvascular networks. Heatmap showing relative gene expression in EC networks cultured in Soft, IM, and Stiff matrix conditions. Gene expression values are presented as log2-transformed relative expression levels from qPCR analysis.

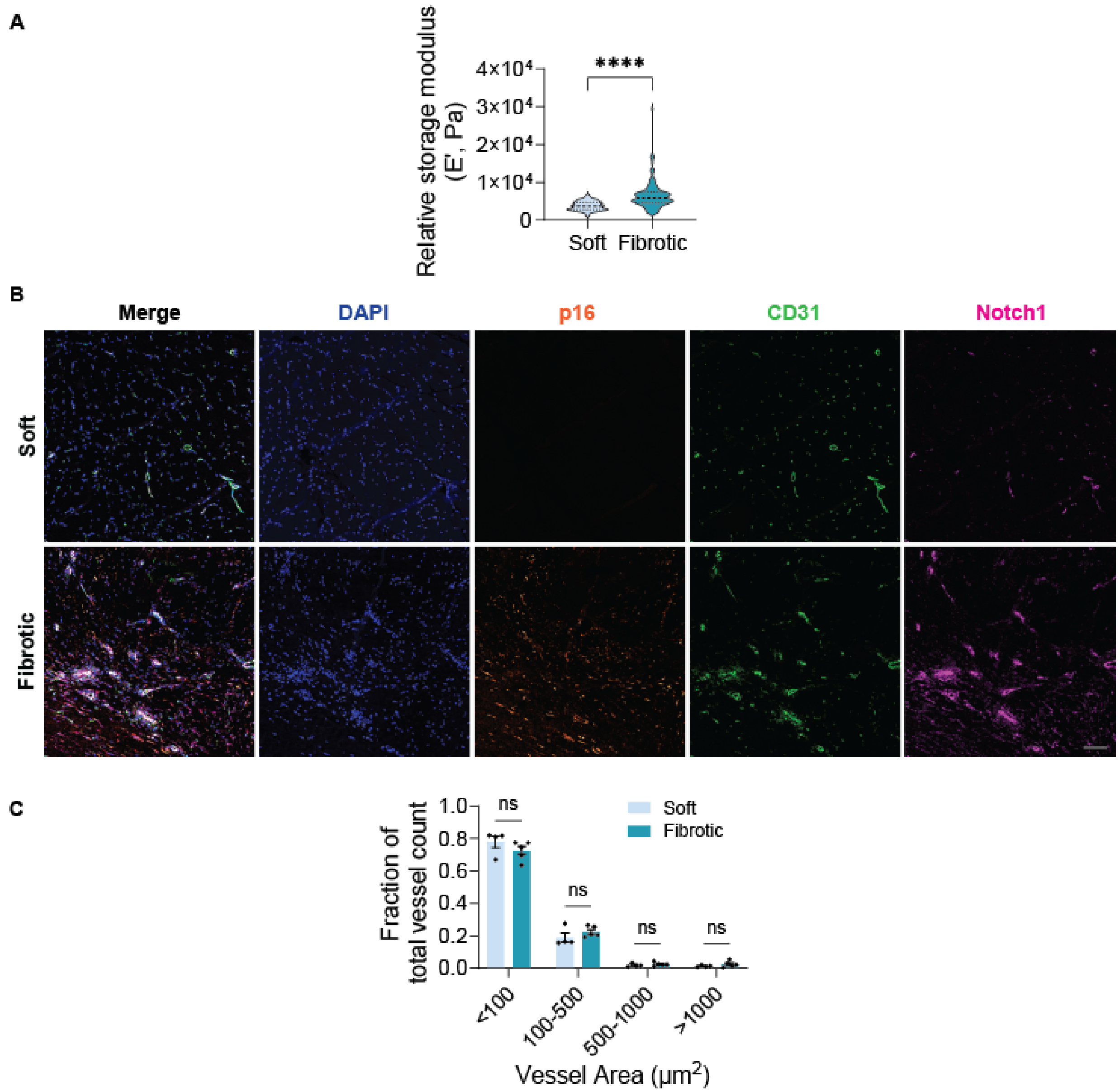

Fig. S5. **Stiffness-associated endothelial senescence and Notch1 activation in human fibrotic capsule tissue.** (A) AFM-based microrheology measurements of human breast implant fibrotic capsule tissues demonstrating increased relative storage modulus in fibrotic regions compared to adjacent non-fibrotic tissues. Due to the use of fixed tissue samples, values are presented as relative mechanical properties. (B) Representative immunofluorescence images of human breast implant capsule tissue, showing DAPI (blue), p16 (senescence marker, orange), CD31 (EC marker, green), and Notch1 (magenta) in soft control and fibrotic regions. Increased p16 and Notch1 signals are observed in fibrotic tissues. Scale bar = 100 μm. (C) Quantification of vessel area distribution in soft and fibrotic tissues. Statistical analyses were performed using Student's t-test. Data are presented as mean ± SEM. Significance levels were set at ****$p \leq 0.0001$.

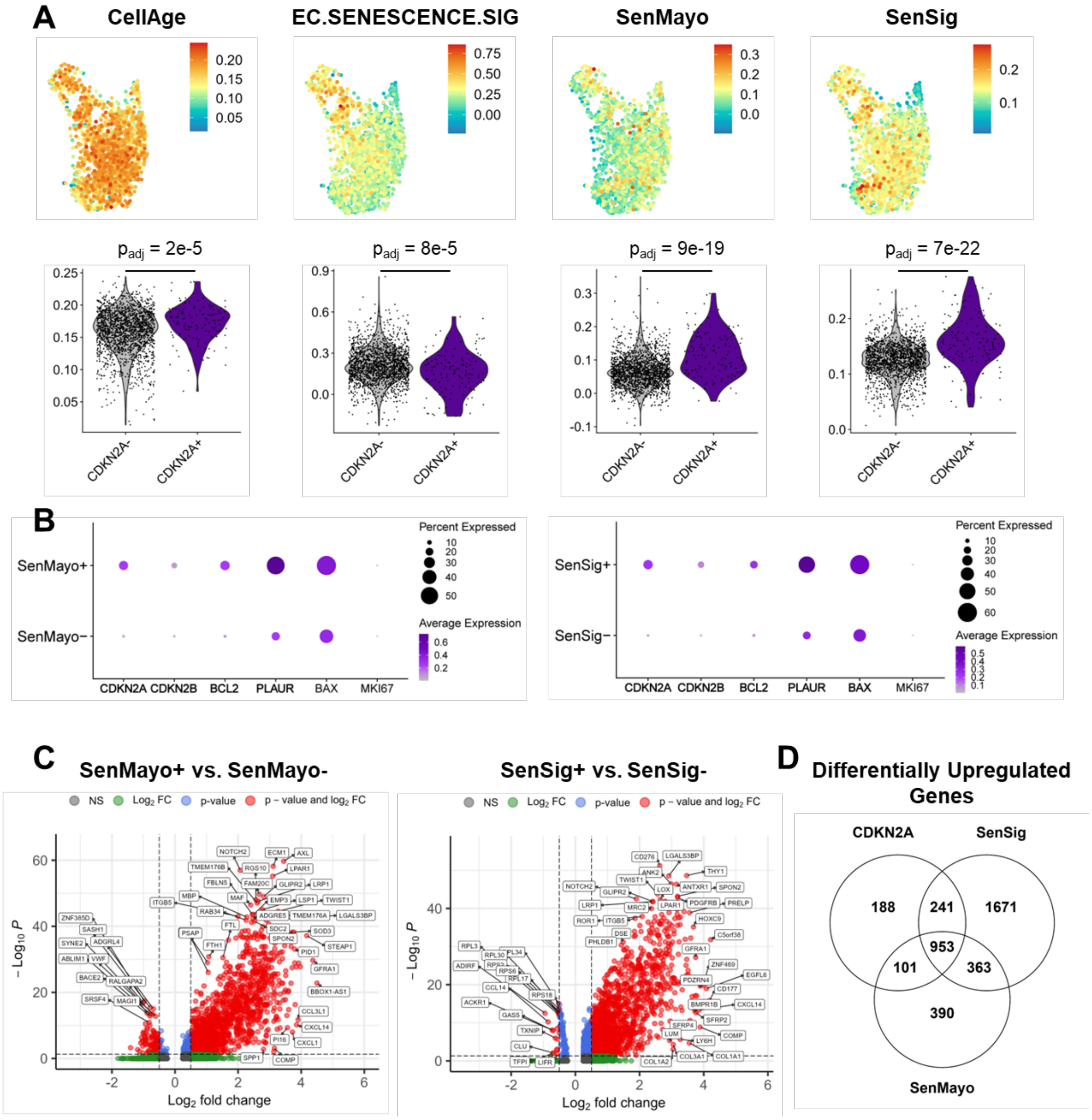

Fig. S6. Comparison of methods to identify senescent ECs using published senescence gene signatures and CDKN2A expression. (A) UMAP projection and violin plot showing the Seurat Module Score of each senescence signature. Violin plots show the signature scores in CDKN2A- and CDKN2A+ ECs. A Wilcoxon Rank Sum Test with Benjamini-Hochberg correction was used to compare signature scores between CDKN2A^{+} and CDKN2A^{-} cells. (B) Dot plots showing expression of senescence-associated genes in senescent and non-senescent ECs using the SenMayo (left) and SenSig (right) classification methods. Dot color represents average normalized expression and dot size represents percentage of cells expressing the gene. Genes shown in bold text are differentially expressed between senescent and non-senescent ECs (p_{adj}<0.05). (C) Volcano plots showing differentially expressed genes between SenMayo+ and

SenMayo- ECs (left) and SenSig+ and SenSig- cells (right). (D) Venn diagram showing the number of shared and unique differentially upregulated genes between the analyses using CDKN2A, SenMayo, and SenSig for senescent cell identification.

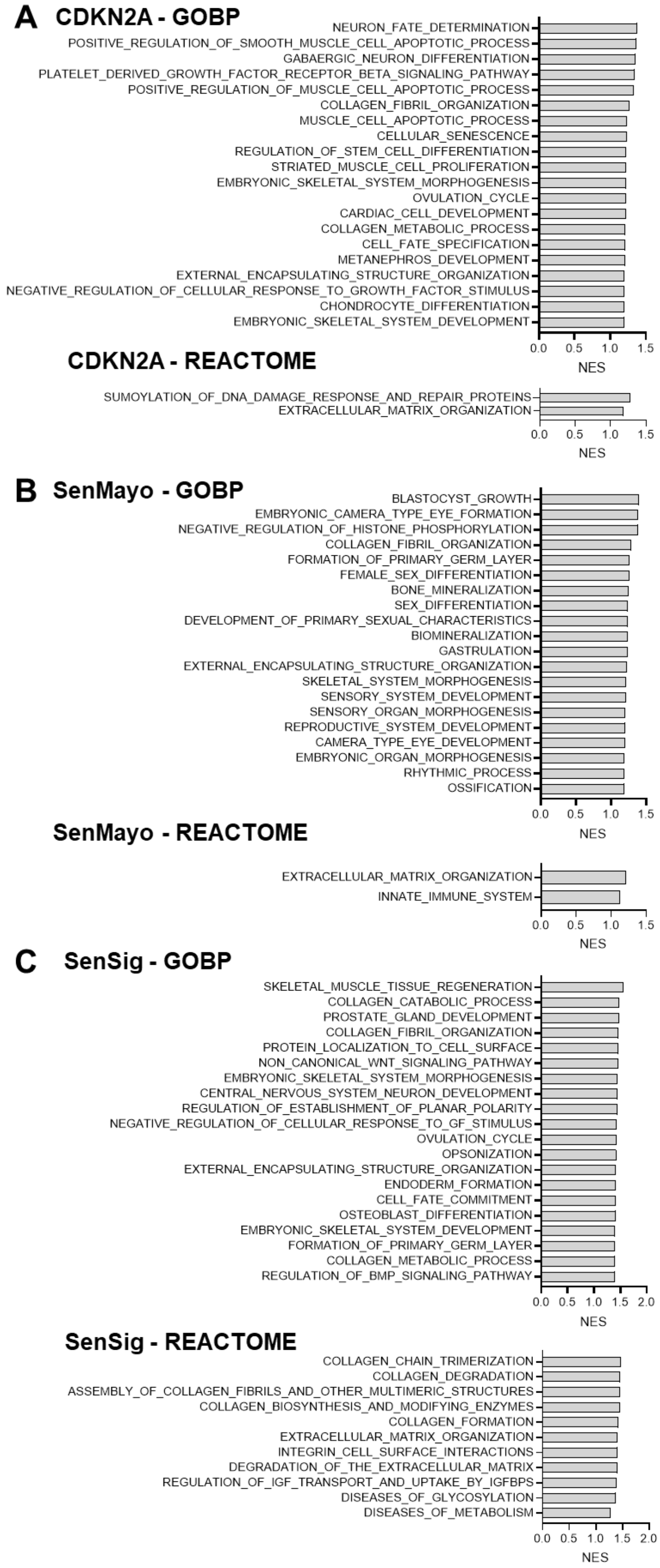
A
CDKN2A - GOBP
NEURON_FATE_DETERMINATION
POSITIVE_REGULATION_OF_SMOOTH_MUSCLE_CELL_APOPTOTIC_PROCESS
GABAERGIC_NEURON_DIFFERENTIATION
PLATELET_DERIVED_GROWTH_FACTOR_RECEPTOR_BETA_SIGNALING_PATHWAY
POSITIVE_REGULATION_OF_MUSCLE_CELL_APOPTOTIC_PROCESS
COLLAGEN_FIBRIL_ORGANIZATION
MUSCLE_CELL_APOPTOTIC_PROCESS
CELLULAR_SENESCENCE
REGULATION_OF_STEM_CELL_DIFFERENTIATION
STRIATED_MUSCLE_CELL_PROLIFERATION
EMBRYONIC_SKELETAL_SYSTEM_MORPHOGENESIS
OVULATION_CYCLE
CARDIAC_CELL_DEVELOPMENT
COLLAGEN_METABOLIC_PROCESS
CELL_FATE_SPECIFICATION
METANEPHROS_DEVELOPMENT
EXTERNAL_ENCAPSULATING_STRUCTURE_ORGANIZATION
NEGATIVE_REGULATION_OF_CELLULAR_RESPONSE_TO_GROWTH_FACTOR_STIMULUS
CHONDROCYTE_DIFFERENTIATION
EMBRYONIC_SKELETAL_SYSTEM_DEVELOPMENT
0.0 0.5 1.0 1.5
NES
CDKN2A - REACTOME
SUMOYLATION_OF_DNA_DAMAGE_RESPONSE_AND_REPAIR_PROTEINS
EXTRACELLULAR_MATRIX_ORGANIZATION
0.0 0.5 1.0 1.5
NES
B
SenMayo - GOBP
BLASTOCYST_GROWTH
EMBRYONIC_CAMERA_TYPE_EYE_FORMATION
NEGATIVE_REGULATION_OF_HISTONE_PHOSPHORYLATION
COLLAGEN_FIBRIL_ORGANIZATION
FORMATION_OF_PRIMARY_GERM_LAYER
FEMALE_SEX_DIFFERENTIATION
BONE_MINERALIZATION
SEX_DIFFERENTIATION
DEVELOPMENT_OF_PRIMARY_SEXUAL_CHARACTERISTICS
BIOMINERALIZATION
GASTRULATION
EXTERNAL_ENCAPSULATING_STRUCTURE_ORGANIZATION
SKELETAL_SYSTEM_MORPHOGENESIS
SENSORY_SYSTEM_DEVELOPMENT
SENSORY_ORGAN_MORPHOGENESIS
REPRODUCTIVE_SYSTEM_DEVELOPMENT
CAMERA_TYPE_EYE_DEVELOPMENT
EMBRYONIC_ORGAN_MORPHOGENESIS
RHYTHMIC_PROCESS
OSSIFICATION
0.0 0.5 1.0 1.5
NES
SenMayo - REACTOME
EXTRACELLULAR_MATRIX_ORGANIZATION
INNATE_IMMUNE_SYSTEM
0.0 0.5 1.0 1.5
NES
C
SenSig - GOBP
SKELETAL_MUSCLE_TISSUE_REGENERATION
COLLAGEN_CATABOLIC_PROCESS
PROSTATE_GLAND_DEVELOPMENT
COLLAGEN_FIBRIL_ORGANIZATION
PROTEIN_LOCALIZATION_TO_CELL_SURFACE
NON_CANONICAL_WNT_SIGNALING_PATHWAY
EMBRYONIC_SKELETAL_SYSTEM_MORPHOGENESIS
CENTRAL_NERVOUS_SYSTEM_NEURON_DEVELOPMENT
REGULATION_OF_ESTABLISHMENT_OF_PLANAR_POLARITY
NEGATIVE_REGULATION_OF_CELLULAR_RESPONSE_TO_GF_STIMULUS
OVULATION_CYCLE
OPSONIZATION
EXTERNAL_ENCAPSULATING_STRUCTURE_ORGANIZATION
ENDODERM_FORMATION
CELL_FATE_COMMITMENT
OSTEOBLAST_DIFFERENTIATION
EMBRYONIC_SKELETAL_SYSTEM_DEVELOPMENT
FORMATION_OF_PRIMARY_GERM_LAYER
COLLAGEN_METABOLIC_PROCESS
REGULATION_OF_BMP_SIGNALING_PATHWAY
0.0 0.5 1.0 1.5 2.0
NES
SenSig - REACTOME
COLLAGEN_CHAIN_TRIMERIZATION
COLLAGEN_DEGRADATION
ASSEMBLY_OF_COLLAGEN_FIBRILS_AND_OTHER_MULTIMERIC_STRUCTURES
COLLAGEN_BIOSYNTHESIS_AND_MODIFYING_ENZYMES
COLLAGEN_FORMATION
EXTRACELLULAR_MATRIX_ORGANIZATION
INTEGRIN_CELL_SURFACE_INTERACTIONS
DEGRADATION_OF_THE_EXTRACELLULAR_MATRIX
REGULATION_OF_IGF_TRANSPORT_AND_UPTAKE_BY_IGFBPS
DISEASES_OF_GLYCOSYLATION
DISEASES_OF_METABOLISM
0.0 0.5 1.0 1.5 2.0
NES

Fig. S7. Gene set enrichment analysis reveals pathways upregulated in senescent ECs classified by CDKN2A (A), SenMayo (B), or SenSig (C). Pathways with a positive normalized enrichment score (NES) and $p_{adj}<0.05$ are displayed. The top 20 pathways by NES are shown for the analyses using the GOBP database. All significant pathways are shown for the analyses using the REACTOME database.

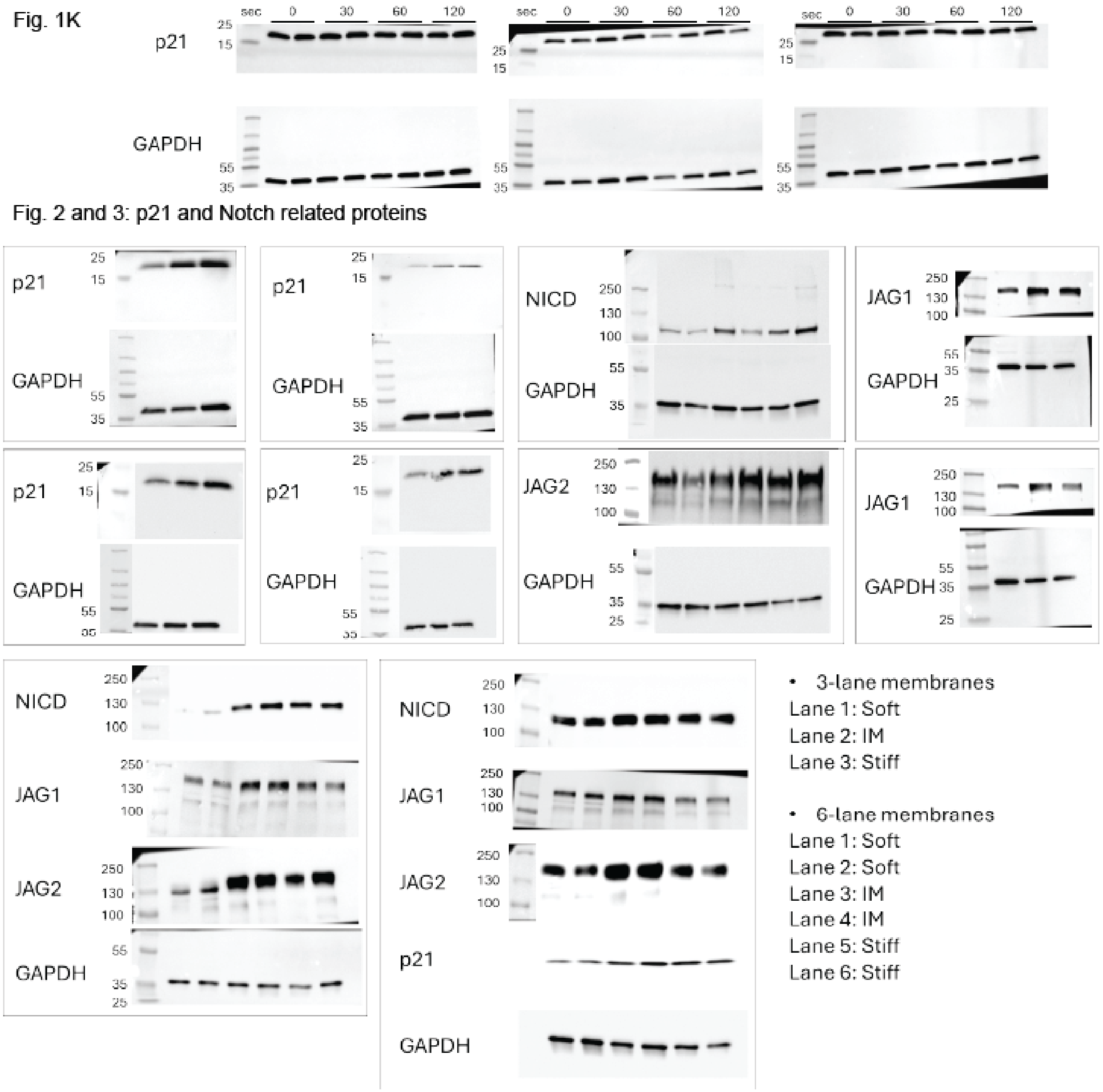

Fig. S8. Western blot membranes corresponding to the main figures are shown.

Fig. 3: JNK and c-JUN proteins

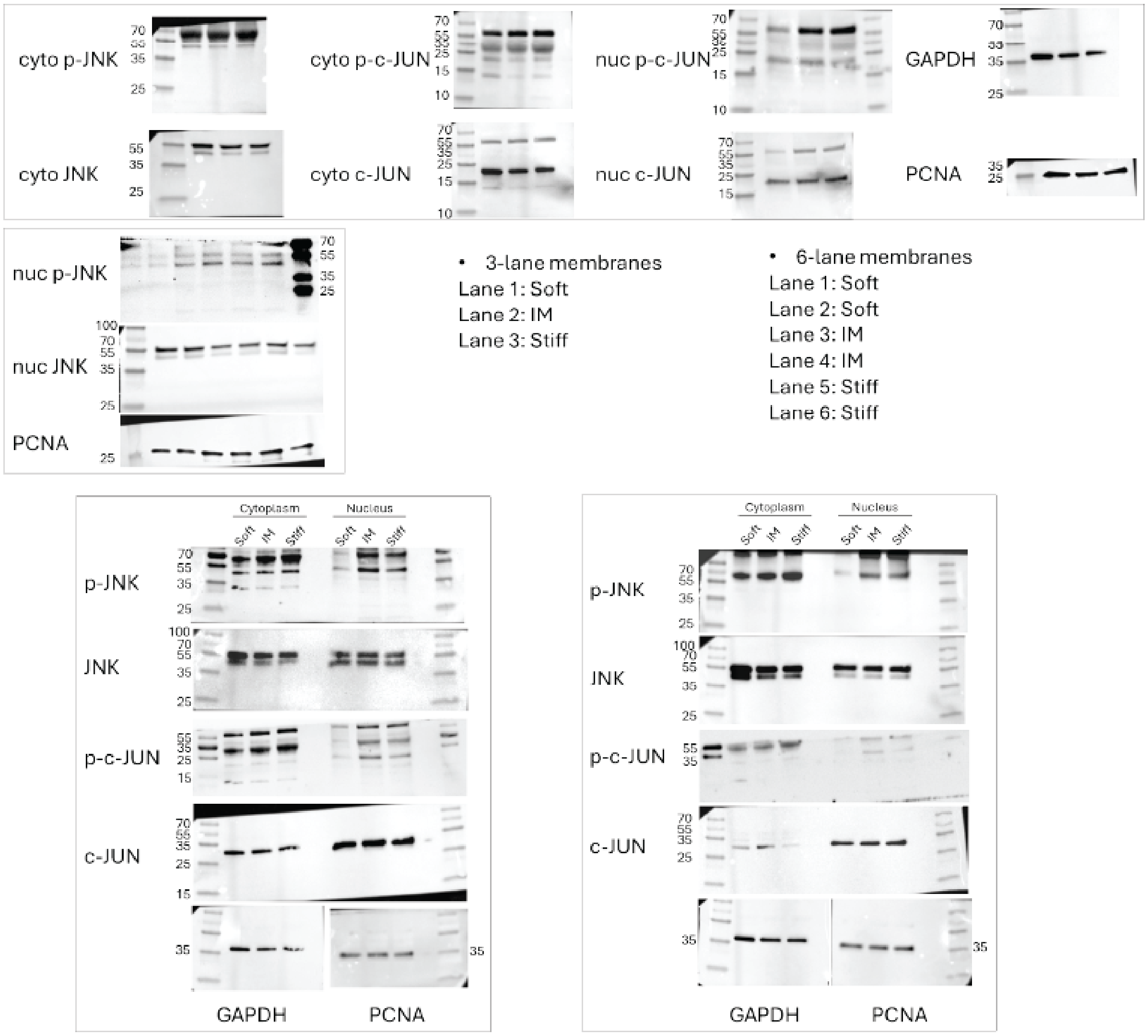

Fig. S8 (continued). Western blot membranes corresponding to the main figures are shown.

Supplementary Tables:

Table S1. Summary of hydrogel formulations and secondary crosslinking parameters used to model dynamic matrix stiffening. Conditions vary by methacrylation level and light exposure to achieve a tunable range of stiffness, as reflected in Young's modulus measurements.

	Collagen-MA Conc. (mg/mL)	HA-MA Conc. (mg/mL)	Degree of Methacrylate Substitution on HA (%)	Photoinitiator Exposure on Day 2	Light Exposure on Day 2	Young's Modulus (Pa)	Vascular Networks
**Soft low**	2.5	0.25	14	+	-	179.30 ± 0.92	+++
**Soft high**	2.5	0.25	64	+	-	191.17 ± 18.58	+++
**IM**	2.5	0.25	14	+	+	274.26 ± 5.72	++
**Stiff**	2.5	0.25	64	+	+	408.32 ± 11.02	+

Table S2. Luminex analytes with reliable detection.

**Analytes (pg/ng)**	Soft 1	Soft 2	Soft 3	Soft 4	Soft 5	IM1	IM2	IM3	IM4	IM5	Stiff 1	Stiff 2	Stiff 3	Stiff 4	Stiff 5
IL-6	4.46	4.21	6.24	6.79	7.12	3.06	2.75	4.60	4.22	4.28	1.62	1.49	4.06	3.92	3.67
IL-33	0.22	0.21	0.24	0.26	0.19	0.39	0.39	0.44	0.35	0.44	0.35	0.42	0.47	0.43	0.43
MMP-3	0.99	1.09	1.06	1.09	1.04	1.72	1.71	1.88	1.78	1.76	1.71	1.80	1.81	1.65	1.77
IL-8/CXCL8	16.00	18.97	35.51	22.89	52.35	3.06	3.00	4.28	4.74	3.09	2.44	2.89	3.00	3.83	2.82
MMP-1	590.51	618.35	711.91	541.73	OOR (High) >	1179.40	1315.66	1564.58	1526.69	1298.50	1296.49	1460.43	1341.30	1733.69	1461.17
CCL2/JE/MCP-1	4.90	4.48	4.16	5.10	5.95	4.74	5.22	8.39	7.24	6.65	5.39	6.19	7.71	7.65	6.53
IFN-gamma	0.12	0.13	0.13	0.13	0.12	0.27	0.30	0.26	0.28	0.26	0.32	0.29	0.26	0.26	0.28
CCL3/MIP-1 alpha	2.11	2.29	2.13	2.12	1.99	4.97	4.55	4.39	4.26	4.84	4.42	4.34	4.91	4.71	5.22
IL-1 alpha/IL-1F1	0.13	0.13	0.13	0.14	0.13	0.22	0.26	0.27	0.29	0.21	0.23	0.22	0.24	0.30	0.26
RAGE/AGER	6.51	6.51	6.68	6.62	6.47	13.63	15.94	15.13	15.38	14.67	14.29	14.76	14.42	14.96	15.62
CCL26/Eotaxin-3	0.15	0.15	0.14	0.13	0.13	0.27	0.30	0.28	0.27	0.28	0.26	0.26	0.30	0.30	0.25
Osteopontin/OPN	23.16	23.57	23.71	22.98	26.70	43.76	41.25	49.34	43.79	34.22	35.38	35.58	32.92	34.07	25.79
ICAM-1/CD54	17.46	16.02	24.43	21.96	21.59	17.14	18.61	19.48	22.16	19.26	17.27	17.17	18.23	19.60	18.29
GDF-15	32.33	29.24	28.82	29.03	29.01	65.24	64.09	67.74	60.36	57.74	44.78	48.02	54.26	59.67	45.31
IL-11	51.92	45.11	62.58	59.16	79.02	62.07	56.25	64.11	60.77	63.49	61.95	57.40	57.97	58.02	57.59
CXCL1/GRO alpha/KC/CINC-1	23.44	21.26	33.81	31.58	42.29	7.56	6.89	9.98	9.53	9.24	4.99	5.78	8.67	8.45	7.09

Table S3. Luminex analytes excluded due to incomplete detection.

**Analytes (pg/ng)**	Soft 1	Soft 2	Soft 3	Soft 4	Soft 5	IM1	IM2	IM3	IM4	IM5	Stiff 1	Stiff 2	Stiff 3	Stiff 4	Stiff 5
TNF-alpha	< OOR (Low)	< OOR (Low)	< OOR (Low)	< OOR (Low)		< OOR (Low)	< OOR (Low)	< OOR (Low)	< OOR (Low)		< OOR (Low)	< OOR (Low)	< OOR (Low)	< OOR (Low)	
IL-17/IL-17A	< OOR (Low)	< OOR (Low)	< OOR (Low)	< OOR (Low)		< OOR (Low)	< OOR (Low)	< OOR (Low)	< OOR (Low)		< OOR (Low)	< OOR (Low)	< OOR (Low)	< OOR (Low)	
IL-13	< OOR (Low)	< OOR (Low)	< OOR (Low)	< OOR (Low)		< OOR (Low)	< OOR (Low)	< OOR (Low)	< OOR (Low)		< OOR (Low)	< OOR (Low)	< OOR (Low)	< OOR (Low)	
VCAM-1/CD106	< OOR (Low)	< OOR (Low)	< OOR (Low)	< OOR (Low)		< OOR (Low)	< OOR (Low)	< OOR (Low)	< OOR (Low)		< OOR (Low)	< OOR (Low)	< OOR (Low)	< OOR (Low)	
TGF-alpha	< OOR (Low)	< OOR (Low)	< OOR (Low)	< OOR (Low)		< OOR (Low)	< OOR (Low)	< OOR (Low)	< OOR (Low)		< OOR (Low)	< OOR (Low)	< OOR (Low)	< OOR (Low)	
CCL8/MCP-2	0.53	0.54	< OOR (Low)	0.56	0.63	1.38	1.30	< OOR (Low)	< OOR (Low)	< OOR (Low)	< OOR (Low)	< OOR (Low)	< OOR (Low)	< OOR (Low)	< OOR (Low)
CXCL2/GRO beta/MIP-2/CINC-3	0.62	0.66	0.68	0.66	0.76	< OOR (Low)	< OOR (Low)	1.11	< OOR (Low)	< OOR (Low)	< OOR (Low)	< OOR (Low)	< OOR (Low)	< OOR (Low)	< OOR (Low)
IL-1 beta/IL-1F2	0.24	0.21	0.23	0.26	0.23	< OOR (Low)	< OOR (Low)	0.50	< OOR (Low)	< OOR (Low)	< OOR (Low)	0.56	0.50	< OOR (Low)	0.50
VEGF-A	OOR (High) >	OOR (High) >	OOR (High) >	OOR (High) >	OOR (High) >	OOR (High) >	OOR (High) >	OOR (High) >	OOR (High) >	OOR (High) >	OOR (High) >	OOR (High) >	OOR (High) >	OOR (High)>	OOR (High) >
Serpin E1/PAI-1	OOR (High) >	OOR (High) >	OOR (High) >	OOR (High) >	OOR (High) >	OOR (High) >	OOR (High) >	OOR (High) >	OOR (High) >	OOR (High) >	OOR (High) >	OOR (High) >	OOR (High) >	OOR (High)>	OOR (High) >

Table S4. Forward and reverse primers used for qPCR analysis.

Gene	Forward Primer (5'-3")	Reverse Primer (5'-3")
GAPDH	ACAACTTTGGTATCGTGGAAGG	GCCATCACGCCACAGTTTC
CDKN1A	ACTCTCAGGGTCGAAAACGG	GATGTAGAGCGGGCCTTTGA
CDKN2A	ATGGAGCCTTCGGCTGACTGGC	CTGCCCATCATCATGACCTGGA
Notch1	CACTGTGGGCGGGTCC	GTTGTATTGGTTCGGCACCAT
JAG1	GACTCATCAGCCGTGTCTCA	TGGGGAACACTCACACTCAA
JAG2	GGTCGTACTTGCACTCACAATACC	GTAGCAAGGCAGAGGGTTGC
HEY1	GCTGGTACCCAGTGCTTTTGAG	TGCAGGATCTCGGCTTTTTCT
Dll4	GAAGTGGACTGTGGCCTGGACAAGT	TCGCTGATATCCGACACTCTGGCT
JUN	CCTTGAAAGCTCAGAACTCGGAG	TGCTGCGTTAGCATGAGTTGGC
MMP2	CTCATCGCAGATGCCTGGAA	TTCAGGTAATAGGCACCCTTGAAGA
COL4A1	TGTTGACGGCTTACCTGGAGAC	GGTAGACCAACTCCAGGCTCTC
COL3A1	GGAGCTGGCTACTTCTCGC	GGGAACATCCTCCTTCAACAG
FN1	CGGTGGCTGTCAGTCAAAG	AAACCTCGGCTTCCTCCATAA
LOX	CGGCGGAGGAAAACTGTCT	TCGGCTGGGTAAGAAATCTGA
